# Cancer Neoaxonogenesis: Mechanisms and Factors Involved in the Recruitment of Peripheral Nerves by Cancer Tissue

**DOI:** 10.3390/ijms27093792

**Published:** 2026-04-24

**Authors:** Filip Blasko, Lubica Horvathova, Luba Hunakova, Lucia Krivosikova, Monika Burikova, Bozena Smolkova, Sara Durdiakova, Benjamin Spanik, Michal Mego, Pavel Babal, Boris Mravec

**Affiliations:** 1 Institute of Physiology, Faculty of Medicine, Comenius University Bratislava, 813 72 Bratislava, Slovakia; boris.mravec@fmed.uniba.sk; 2 Institute of Experimental Endocrinology, Biomedical Research Center, Slovak Academy of Sciences, 845 05 Bratislava, Slovakia; lubica.horvathova@savba.sk; 3 Institute of Immunology, Faculty of Medicine, Comenius University Bratislava, 811 08 Bratislava, Slovakia; luba.hunakova@fmed.uniba.sk; 4 Institute of Pathological Anatomy, Faculty of Medicine, Comenius University Bratislava, 811 08 Bratislava, Slovakia; lucia.krivosikova@fmed.uniba.sk (L.K.); pavel.babal@fmed.uniba.sk (P.B.); 5 Department of Molecular Oncology, Institute of Experimental Oncology, Biomedical Research Center, Slovak Academy of Sciences, 845 05 Bratislava, Slovakia; monika.burikova@savba.sk (M.B.); bozena.smolkova@savba.sk (B.S.); 6 2nd Department of Oncology, Faculty of Medicine, Comenius University Bratislava, National Cancer Institute, 833 10 Bratislava, Slovakia; durdiakova6@uniba.sk (S.D.); spanik17@uniba.sk (B.S.); michal.mego@nou.sk (M.M.)

**Keywords:** axonal guidance molecules, cancer progression, exosomes, neoaxonogenesis, neurobiology of cancer, neurotrophic factors

## Abstract

Peripheral nerves provide a direct connection between the brain and the tumor microenvironment. This connection allows the nervous system to influence processes associated with the development, progression, and metastasis of different tumor types. Therefore, tumor innervation by peripheral nerve fibers is currently emerging as a characteristic that contributes to multiple hallmarks of cancer. Several experimental studies have shown that cancer progression involves actively inducing the ingrowth of autonomic and sensory nerve fibers into tumor tissue. In this process, known as neoaxonogenesis, cancer and other cells in the tumor microenvironment play an important role by synthesizing and releasing neurotrophic factors (e.g., nerve growth factor, brain-derived neurotrophic factor, glial cell line-derived neurotrophic factor), axonal guidance molecules (netrins, semaphorins, ephrins, slits), exosomes (containing microRNA and axonal guidance molecules), and other molecules present in the tumor microenvironment (e.g., granulocyte colony-stimulating factor, leukemia inhibitory factor), which modulate the ingrowth of nerve fibers into the tumor. This results in an increased nerve supply to tumor tissue, which is primarily linked to its growth. However, there are also studies demonstrating the protective effects of increased nerve fiber density against processes associated with cancer progression in certain types of cancer. The findings from these studies contribute to the complexity of neuro-cancer interactions, which is probably based on the type of cancer and the physiological specializations of the nerve fibers in a given organ. Despite contrasting findings, the stimulatory effects of nerve fibers on cancer growth are supported by several studies that described reducing the negative impact of nerve fibers on tumors and thus inhibiting cancer progression. The most significant approaches to reducing neural effects appear to be denervation, the administration of neurotransmitter receptor antagonists, the administration of local anesthetics, and the administration of antibodies against neurotrophic factors. Other significant approaches include methods that improve quality of life, such as psychotherapy and heart rate variability biofeedback. Despite their therapeutic potential, there are several limitations to using approaches that manipulate cancer innervation in clinical practice. These limitations include impaired normal tissue function and nervous system function, as well as the problematic direct application of the therapeutic agent to the tumor site, dosage-dependent, cancer type-dependent, cancer stage-dependent, duration-dependent, and timing-dependent effects. Procedures that modify neoaxonogenesis and nerve fiber signaling appear to be a promising new therapeutic approach in oncology. However, more research is needed to better understand their effects on cancer progression. In the future, the assessment of the presence and density of nerve fibers in tumors, as well as the evaluation of approaches aimed at reducing their negative impact, could be part of personalized anticancer therapy. As part of this therapy, a fresh tumor sample would be collected from the patient to generate patient-derived organoid models to test and consider the possibility of using supportive therapy and to predict its efficacy. Based on these results, it would be possible to evaluate the applicability of nerve-fiber-targeted therapy for a given patient. This review article summarizes and describes the current knowledge concerning the significance of nerve fibers in cancer progression, with a particular emphasis on neoaxonogenesis in tumors and the various factors that influence this process.

## 1. Introduction

Research on the role of the nervous system in cancer has greatly expanded our knowledge of the mechanisms that lead to tumor development. However, the complex etiopathogenesis of cancer is not fully understood. A significant finding was the discovery that peripheral tumor tissues, i.e., the microenvironment of solid and hematological malignancies located outside the nervous system, are innervated by peripheral nerve fibers. Various neurotransmitters and neuromodulators are released from the nerve endings of these nerves and modulate signaling pathways associated with tumor development, progression, and metastasis [1,2]. Therefore, nerve fibers in tumors are not just a rudimentary element, but they significantly influence the progression of tumors and metastasis. For this reason, tumor nerve fiber density is considered a significant prognostic factor, as higher levels are typically associated with poorer clinical outcomes and shorter patient survival [2,3,4,5].

Nerve fibers become part of the tumor tissue through various mechanisms. One important mechanism is the ingrowth of nerve fibers (neoaxonogenesis) from surrounding nerves into tumors in response to the production and release of neurotrophic factors, axonal guidance molecules, and exosomes by cancer cells [6,7,8]. This results in increased neural influence on the tumor microenvironment, which is primarily associated with stimulating cancer cell proliferation and migration, inducing neoangiogenesis, and modulating immune cell functions [9,10,11]. The importance of tumor innervation is supported by clinical and experimental findings, which suggest that approaches limiting the effects of nerve fiber signaling pathways on tumors (e.g., surgical or chemical denervation, administration of neurotransmitter receptor antagonists, local anesthetics, antibodies against neurotrophic factors) or approaches that generally reduce the negative effects of stress (e.g., psychotherapy, mindfulness techniques and heart rate variability biofeedback) can inhibit tumor progression and improve patient outcomes [1,6,12,13,14,15,16]. On the other hand, it should be noted that nerve fibers may not only stimulate the cancer growth, but they may also promote repair and regeneration processes in tissues, thereby acting as a protective mechanism against tumor development and progression [3,17,18,19]. Recent data suggests that nerve types can promote or inhibit tumor progression, likely based on the type of receptors expressed by cells in the tumor microenvironment, the degree of immunosuppression in tumors, tumor type, tumor localization, and the physiological specialization of nerve fibers in a given organ (e.g., the stimulatory effects of parasympathetic nerves in gastric and colorectal cancers). To clarify which factors influence nerve types to stimulate or inhibit tumor growth, longitudinal and tumor-type-specific studies will be required, as well as tools that can selectively manipulate one neural subtype. Although the findings are inconclusive, studying cancer innervation and the mechanisms by which nerve fibers influence tumors shows great therapeutic potential. This knowledge may contribute to the identification of new therapeutic approaches based on the regulation of neural-tumor interactions, which could improve the effectiveness of antitumor therapy. Based on these facts, tumor innervation is currently accepted as a significant prognostic factor and is emerging as a characteristic affecting multiple hallmarks of cancer [5,20,21]. However, due to the extremely complex and tumor-type-specific influence of nerve fibers on tumors, more research is needed to better understand their contribution to tumor progression.

This review article summarizes the current knowledge concerning the significance of nerve fibers in cancer progression, with a particular emphasis on neoaxonogenesis in tumor tissue and the molecules and factors that modulate this process. The individual chapters and subsections provide detailed descriptions of tumor innervation sources, summarize findings on the roles of autonomic and sensory nerve fibers in tumor progression and metastasis, and discuss approaches to reducing the negative effects of nerve fibers on tumors. Additionally, limitations of approaches targeting nerve fibers and future challenges in the neurobiology of cancer research are described, as well. This paper provides a comprehensive view of the process of neoaxonogenesis in tumors and describes the modulation of neoaxonogenesis by neurotrophic factors, axonal guidance molecules, and exosomes. These factors are usually described separately or to a much lesser extent. This paper summarizes the latest and most significant findings in this field across various tumor types and experimental approaches. Additionally, this paper critically evaluates the most significant, potentially applicable, supportive therapeutic approaches, describing their benefits and the significant limitations that must be thoroughly investigated before they can be widely applied in clinical practice. We believe this paper will provide researchers and clinicians with a solid theoretical foundation and a better understanding of nerve fiber ingrowth into tumors and its role in tumor progression. It will also help them better navigate the broad, often inconsistent spectrum of results from interventional studies and serve as a useful source of information when planning further experimental and clinical research.

For this purpose, we searched related original articles and reviews using the PubMed database by use of search terms: “cancer”, “tumor”, “innervation”, “axonogenesis”, “adrenergic”, “cholinergic”, “nervous system”, “neurotransmitters”, “stress”, “sympathetic”, “parasympathetic”, “vagal”, and “sensory”. In general, we avoided using preprint articles due to the unfinished peer review process. However, we included one preprint article due to its relevance to the discussed topic. Only papers published in English between 2000 and January 2026 were included.

## 2. Tumor Innervation

### 2.1. Physiological Organization of the Peripheral Nervous System

The nervous system is the main control and integration system of the body, which consists of a complex, interconnected network of neurons and glial cells, as well as their projections [10,22]. The nervous system is anatomically divided into two parts: the central nervous system (CNS) and the peripheral nervous system (PNS). The CNS consists of two structures that regulate bodily functions: the brain and the spinal cord [23,24]. The PNS is divided into two divisions based on function: the sensory (afferent) division and the motor (efferent) division. The sensory (afferent) division is further subdivided into two systems: the somatic sensory system, which includes somatic mechanoreceptors, nociceptors, thermoreceptors, and proprioceptors, and the visceral sensory system, which includes mechanoreceptors, nociceptors, and other receptors in visceral organs. This component of the PNS is responsible for the transmission of information from receptors to the CNS. The motor (efferent) division is classified into the somatic motor system and the visceral motor system (equivalent to the autonomic nervous system). The somatic motor system is responsible for relaying signals from the CNS to the skeletal muscles, responsible for inducing their voluntary contraction. The visceral motor system, or the autonomic nervous system (ANS), is responsible for the regulation of physiological functions in visceral organs, blood vessels, exocrine and endocrine glands (e.g., cardiac muscle activity, respiration, digestion, metabolic functions, excretion, sexual functions, glandular secretion, immune functions, and others), which is necessary for maintaining homeostasis. The ANS is divided into four parts based on its morphological and functional characteristics: the sympathetic, parasympathetic, enteric, and intracardial ANS [24].

All human tissues, except for cartilage and the lens, are innervated by sensory (afferent) and motor (efferent) nerve fibers. These fibers enable active movement, sensation of stimuli from the environment, tissue repair and regeneration, and physiological regulation of visceral organs [10,24,25]. Under physiological conditions, nerve fibers play a crucial role in maintaining homeostasis in the body. However, several studies have shown that they also play a significant role in the development and progression of diseases, including cancer [10,22].

### 2.2. The Origin and Presence of Nerve Fibers in Peripheral Tumors

The microenvironment of peripheral tumors (i.e., tumors primarily located outside the nervous system, such as breast, prostate, colorectal, and pancreatic cancers) is innervated by sympathetic, parasympathetic, and sensory nerve fibers from peripheral nerves, depending on the location of organs with neoplastic changes. These nerves are an integral part of the tissues and organs in which malignant transformation of normal cells into cancer cells has occurred [1,26]. The axonal terminals of nerve fibers in tumors interact directly with cancer cells and other cells in the tumor microenvironment by creating pseudo-synaptic connections and/or releasing neurotransmitters and neuromodulators. These substances can directly stimulate processes associated with tumor development, progression, and metastasis by binding to receptors on cancer cells and other cells in the tumor microenvironment [13,27,28].

**Figure 1:**
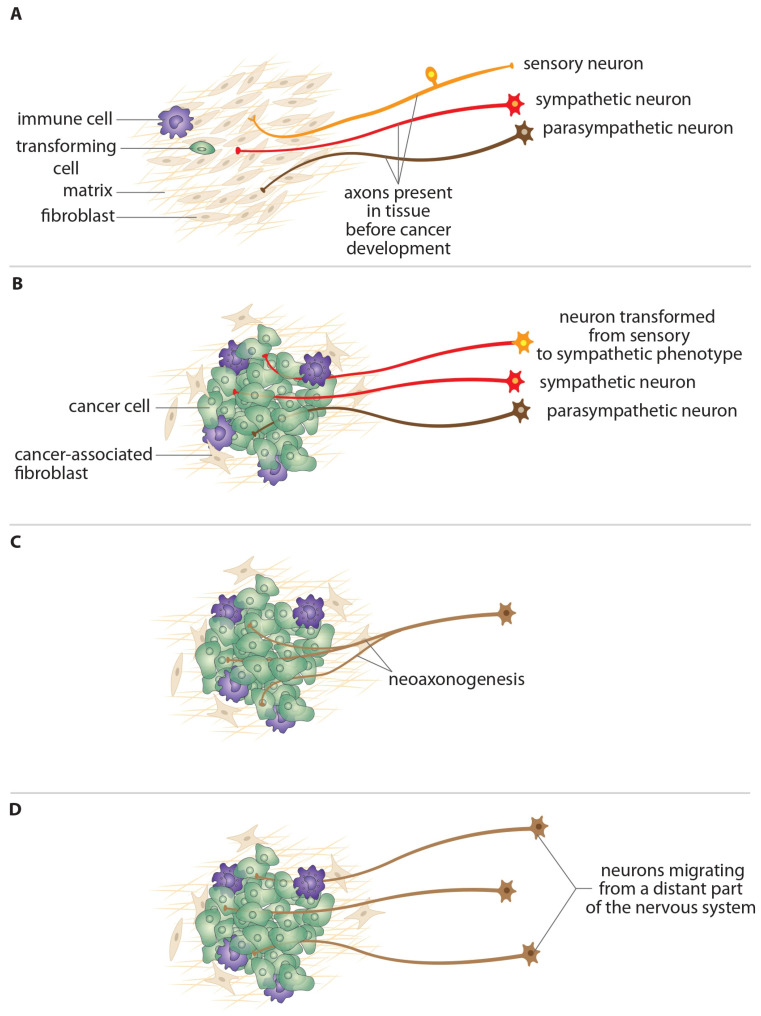
Schematic depiction of the origin of cancer innervation. Nerve fibers that innervate tumor tissues originate from various sources. (**A**)—autonomic (sympathetic, red; parasympathetic, dark brown) and sensory (orange) nerve fibers present in the normal tissue prior to the malignant transformation of its cells into cancer cells; (**B**)—the axons of the phenotypically transformed sensory neurons into sympathetic neurons; (**C**)—newly formed axons that grow into tumor tissue from peripheral nerves located near the tumor (neoaxonogenesis); (**D**)—axons of neurons that have migrated directly into tumor tissue or into the tumor environment from distant parts of the central (CNS) or peripheral nervous system (PNS) (modified according to Mravec [39]).

In general, available data indicate that there are various sources from which tumor-innervating nerve fibers originate (Figure 1):

- Nerve fibers that were present in the normal tissue prior to the malignant transformation of its cells into cancer cells, which became part of the tumor as a result of cancer cell proliferation and expansive tumor growth [22,29]. However, these nerve fibers are damaged during tumor growth, as demonstrated by Shurin et al. [29] in mouse and human melanoma. This damage triggers the reprogramming of Schwann cells into repair cells. The activated Schwann cells influence the tumor microenvironment by modulating immune cell activity and altering the extracellular matrix, which stimulates tumor growth [29].
- The axons of the phenotypically transformed sensory neurons into sympathetic neurons, which innervated the normal tissue, stimulating processes associated with the growth and spread of cancer cells to other parts of the body [30]. The existence of this mechanism was demonstrated by Amit et al. [30] using a mouse head and neck cancer model. While the original sensory fibers were found to inhibit tumor growth, the potentiation of tumor growth by transdifferentiated adrenergic fibers was observed.
- Newly formed axons that grow into tumor tissue from peripheral nerves located near the tumor (neoaxonogenesis) [6,8,31]. The fact that tumors are not isolated structures in the body and interact with numerous cell types (e.g., stromal fibroblasts, endothelial cells, immune cells), components of the extracellular matrix, tissues, and organ systems to exploit normal developmental processes to drive carcinogenesis has been repeatedly demonstrated. Through these interactions, cancer cells stimulate the formation of new blood vessels (neoangiogenesis) to supply oxygen and nutrients and remove waste products. They also promote the formation of new lymphatic vessels (neolymphangiogenesis), which is associated with the migration of cancer cells and metastasis. Furthermore, cancer cells induce the ingrowth of new nerve fibers into tumor tissue (neoaxonogenesis) [8,31]. In recent years, several mechanisms involved in the regulation of neoaxonogenesis have been characterized in detail in various cancer types, including melanoma, breast, gastric, ovarian, prostate, pancreatic, colorectal, head and neck, and others [32,33,34,35].
- The axons of neurons that have migrated directly into tumor tissue or into the tumor environment from distant parts of the CNS or PNS after its formation [36]. This theory of the origin of tumor innervation has been proposed based on the experiments of Mauffrey et al. [36]. They demonstrated that prostate tumors and metastases in mice were infiltrated by neural progenitor cells (NPCs) that express doublecortin (DCX). These cells migrated from the neurogenic area (the subventricular zone) of the brain, after passing the blood–brain barrier and entering the circulation. Furthermore, they found that these progenitor cells changed their phenotype to become adrenergic (sympathetic) neurons [36]. In the clinical part of this study, they found that the density of DCX^+^ neural progenitor cells in human prostate tumors was closely related to prostate cancer aggressiveness and recurrence. Findings from a study conducted by Trinh et al. [37] demonstrated the presence of DCX^+^ neural progenitor cells in human pancreatic tumors, with an increase in density from low-grade to high-grade cases. Similarly, Bjornstad et al. [38] described the presence of cells co-expressing DCX and neurofilament-light (NFL) in the stroma of human breast tumor tissue samples. In an in vitro study, they also found that co-incubation of breast tumor cells and neural progenitors contributed to a more aggressive breast cancer phenotype. Despite the findings of these studies, questions remain about which signaling molecules (microRNAs or others) are responsible for activating brain progenitor cells, guiding them to the tumor site, and transforming them into an adrenergic phenotype. Additionally, it is unclear whether this mechanism is present in other tumor types besides prostate, pancreatic, and breast cancer.

### 2.3. Tumor Neoaxonogenesis, Neoneurogenesis, and Perineural Invasion

In the context of studying tumor innervation, it is necessary to distinguish between several different but interconnected mechanisms that have been described, including neoaxonogenesis, neoneurogenesis, and perineural invasion (PNI) [8,36,40,41]. Neoaxonogenesis refers to the process by which cancer cells induce the active growth of new nerve fibers into the tumor microenvironment from surrounding nerves. This process is regulated by several molecules and factors, which will be described in the following chapters. Neoneurogenesis reflects the infiltration of the tumor microenvironment by neurons that migrate from the CNS or PNS, as well as the formation of new neurons in tumors by the transdifferentiation of tumor stem cells or cancer cells into cells with a neuronal phenotype [36,37,38]. Perineural invasion (PNI) occurs when cancer cells invade the perineural space of peripheral nerves within or around tumor tissue. This process results from interactions between Schwann cells, tumor cells, and axons. PNI is associated with a poor prognosis because it provides a route for cancer cell dissemination, increases the risk of tumor recurrence, and stimulates cancer neoaxonogenesis, which can, in turn, stimulate tumor growth. During PNI, Schwann cells play a dominant role by interacting directly with cancer cells through plasma membrane proteins and indirectly via secretory proteins. After contacting cancer cells, the Schwann cells intercalate between them, stimulating tumor protrusion and dispersion. This process promotes the migration and invasion of cancer cells along nerves [42]. PNI has been documented in several types of cancer that are characterized by a high density of innervation even under physiological circumstances, such as pancreatic, gastric, head and neck, prostate, colorectal, and cervical cancers [40,43,44].

### 2.4. The Role of Autonomic and Sensory Nerves in Cancers

**Figure 2:**
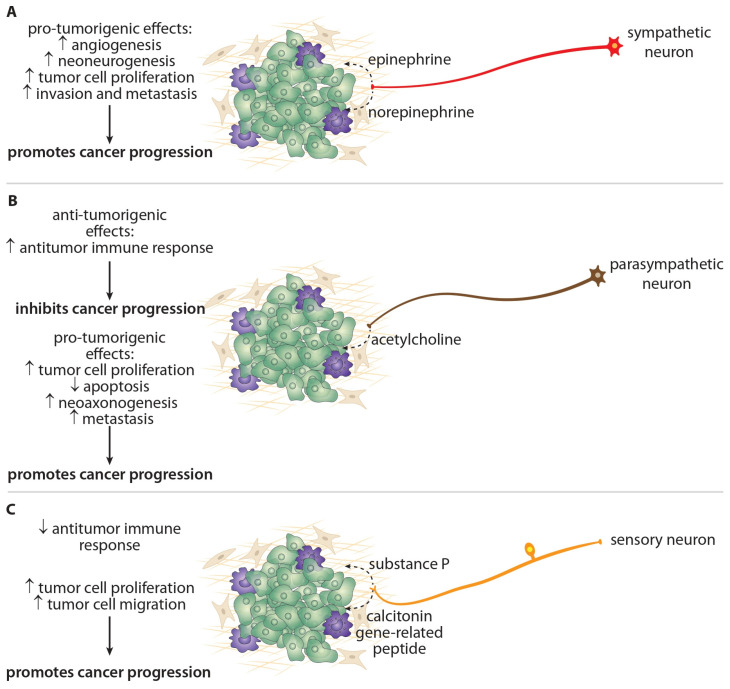
Cancer innervation-mediated effects on cancer progression. The tumor microenvironment is innervated by sympathetic ((**A**), red), parasympathetic ((**B**), dark brown), and sensory ((**C**), orange) nerve fibers based on its location. The axonal nerve endings of intratumoral nerve fibers interact with tumor cells and other cells in the tumor microenvironment by creating pseudo-synaptic structures and/or releasing neurotransmitters and neuromodulators. These interactions modulate processes associated with tumor initiation, progression, and metastasis by activating receptor signaling pathways. (**A**)—the effects of the sympathetic nervous system (red) on cancer are mediated by two neuromediators: epinephrine, which is released from the adrenal medulla and transported to the tumor microenvironment via the bloodstream; and norepinephrine, which is released from intratumoral sympathetic nerve endings. Epinephrine and norepinephrine mediate pro-tumorigenic effects by binding to β-adrenergic receptors, especially β_2_-adrenergic receptors, and activating the adenylate cyclase-cyclic adenosine monophosphate (cAMP) pathway. These effects include stimulation of neoangiogenesis, cancer cell proliferation, migration, tumor metastasis, neoneurogenesis, and neoaxonogenesis in tumors, and also modulation of immune cell functions. (**B**)—the effects of the parasympathetic nervous system (dark brown) are mediated via acetylcholine-mediated activation of nicotinic and muscarinic signaling, which exhibit both anti-tumorigenic and pro-tumorigenic effects, based on the type of tumor. The anti-tumorigenic effects are mediated by promoting an anti-tumor immune response. Conversely, pro-tumorigenic effects are mediated by stimulating cancer cell proliferation, inhibiting apoptosis, promoting neoangiogenesis in tumors, and encouraging metastasis. (**C**)—the effects of sensory nerve fibers (orange) are mediated by two neuromediators, which are released as part of the axonal reflex: substance P (after binding to neurokinin receptor 1) and calcitonin gene-related peptide (after binding to the receptor activity-modifying protein-1). These neuromediators can promote cancer progression by directly stimulating cell proliferation and migration, neoangiogenesis, or by regulating the antitumor immune response (modified according to Vermeer et al. [26]).

In the study of cancer innervation, attention has mainly been given to sympathetic autonomic nerve fibers. These nerves are a part of the sympathoadrenal system, which is responsible for the neuroendocrine stress response associated with the release of norepinephrine (NE) from postganglionic sympathetic nerve endings, as well as the release of epinephrine (EPI) and, to a lesser extent, NE from the adrenal medulla [27,45,46]. Epinephrine released from the adrenal medulla is transported via the bloodstream to the tumor microenvironment. NE is released from sympathetic nerve endings that directly innervate the tumor microenvironment, or from the tissues surrounding the tumor. Released EPI and NE bind to β-adrenergic receptors—β-AR (primarily β_2_-AR, but also β_3_-AR), expressed on cancer cells and other cells in the tumor microenvironment, and predominantly mediates the stimulatory effects of sympathetic nerve fibers on cancer cell proliferation and migration, neoangiogenesis, metastasis, and also the modulatory effects on immune system functions in various types of tumors (Figure 2, Table 1) [1,3,45,47].

Although the activity of parasympathetic nerve fibers under physiological conditions is primarily associated with the resting state (e.g., rest, digestion), several studies have shown that they stimulate the progression and metastasis of certain types of tumors, similarly to sympathetic nerve fibers [1,2,13,48,49]. Unlike the predominantly stimulating effect of the sympathetic nervous system, the effects of the parasympathetic nervous system on cancer appear to be less consistent across tumor types. It is important to note that parasympathetic innervation is much more limited than sympathetic and sensory fibers, which are present in all peripheral tissues. For example, parasympathetic innervation is absent from the skin and limbs. Based on the available data, parasympathetic nerve fibers primarily promote the progression of gastrointestinal tract tumors, likely reflecting their physiological functional specialization [34,50,51]. Furthermore, the effect of parasympathetic fibers on cancer is complicated by studies demonstrating their inhibitory effects on tumor progression, which was associated with a better prognosis in patients [18,19]. The effect of parasympathetic nerve fibers on tumors is mediated by acetylcholine released from postganglionic nerve endings after binding to nicotinic or muscarinic receptors on cancer cells (Figure 2, Table 1) [20,26].

Although sensory nerve fibers primarily transmit information from receptors to the CNS, several studies have demonstrated their stimulatory effects on tumors [52,53]. Two neurotransmitters play a particularly important role in mediating the influence of sensory nerves on cancer: substance P (after binding to neurokinin receptor 1—NK-1R) and calcitonin gene-related peptide (after binding to the RAMP1 receptor), which stimulate cancer cell proliferation, neoangiogenesis, cancer cell migration, metastasis, and also suppress antitumor immunity (Figure 2, Table 1) [26,54,55,56,57]. These neurotransmitters are released into tumor tissue as part of the axonal reflex. Similar to parasympathetic nerve fibers, several studies have shown that sensory fibers may also inhibit tumor progression [17,58].

**Table 1:** The impact of different types of nerve fibers (sympathetic, parasympathetic, sensory) on various types of tumors.

Cancer Type	Innervation Type	Nerve Effects on Cancer	Studies
**Breast** **Cancer**	Sympathetic	↑ MDA-231 cancer cell migration and tumor metastasis into the bones in mice with MDA-231-induced tumors through the induction of RANKL expression in bone osteoblasts after NE β_2_-AR signaling	[59]
		↑ metastasis to the lymph node and lungs in mice with MDA-MB-231-, MDA-MB-453-, and MDA-MB-468-induced tumors through NE-activated β_2_-AR signaling	[60,61]
		↑ cancer progression through increased expression of pro-angiogenic factors VEGF and IL-6 in MDA-MB-231 and MDA-MB-231BR cells mediated by β_2_-AR signaling	[62]
		↑ 4T1 cancer cell proliferation and migration through increased phosphorylation of ERK 1/2 mediated by NPYR5 signaling, activated by NPY	[63]
		↑ cancer cell proliferation and tumor growth in cancer patients by β_1_- and β_3_-AR-mediated increased phosphorylation of MAPK and CREB, and decreased phosphorylation of Akt (PKB), GSK3, and p53	[64]
		↑ metastasis in mice with 66cl4-induced tumors through increased tumor infiltration by MDSCs and M2 macrophages, and by increased gene expression of TGF-β, VEGF, ARG-1, M-CSF, COX-2, MMP-9, and IFN-β mediated by β_2_-AR signaling	[45]
	Sensory	↓ cancer metastasis in mice with 4T1-induced tumors through an increased number of T-lymphocytes, the infiltration of tumor tissue by cytotoxic CD8+ T-lymphocytes, increased levels of IFN-γ and IL-10, and decreased levels of IL-6	[58]
		↑ tumor growth and metastasis MCF10A, PY8119 cells in mice with MDA-MB-231-, MDA-MB-453-, MDA-MB-468-, MCF10A-, and PY8119-induced tumors	[61,65]
		↑ tumor growth and metastasis in mice with 4T1-induced tumors, PDX models, and human and mice organoids through ssRNA molecules released from dying cells and acting on tumoral TLR7 receptors after TACR1 signaling, activated by substance P	[66]
**Prostate** **Cancer**	Sympathetic	↑ cancer progression into metastatic NEPC in mice with LASCPC-01-induced tumors and ↑ prevalence of changes associated with NEPC development in DU145, LNCaP cell lines, and human tumor samples induced by NE β_2_-AR signaling	[3]
		↑ neoangiogenesis and cancer progression in mice with PC-3-induced tumors through HDAC2-mediated suppression of neoangiogenesis inhibitor TSP1, induced by β-AR-cAMP-PKA-CREB signaling	[67]
		↑ PC-3 prostate cancer cell proliferation through NPYR1 and NPYR2 signaling, activated by NPY	[68]
		↑ early phases of cancer development in mice with PC-3-induced tumors through increased nerve density mediated by β_2_- and β_3_-AR signaling	[1]
		↑ metastasis in mice with PC-3-induced tumors through NE-activated β_2_-AR signaling	[69]
		↑ human prostate cancer development and progression at early stages through increased NPY and MIC-1 expression	[70]
		↑ neoangiogenesis and tumor growth in mice with PC-3-induced tumors through inhibition of COA6-mediated oxidative phosphorylation in endothelial cells by NE β_2_-AR signaling	[71]
	Parasympathetic	↑ tumor invasion and metastasis in mice with PC-3-induced tumors through increased nerve density mediated by acetylcholine M1R signaling	[1]
		↑ PC-3 and LNCaP prostate cancer cell migration and invasion through hedgehog signaling mediated by acetylcholine M1R signaling	[72]
**Colorectal** **Cancer**	Sympathetic	↑ neoaxonogenesis in mice with RKO-induced tumors through BDNF-TrkB signaling, which is triggered by increased NE levels and β_3_-AR signaling, and subsequent stimulation of tumor growth and metastasis by these nerves	[33]
		↑ HT-29, HCT116, and RKO cancer cell proliferation, migration, and invasion, and stimulation of tumor progression in mice with HCT116-induced tumors and patients with colorectal tumors through increased miR-373 expression mediated by NE β_1_- and β_2_-AR-cAMP-PKA-CREB1 signaling	[73,74,75]
		↑ cancer cell proliferation, tumor progression, and metastasis in colorectal cancer patients via β_2_-AR signaling	[4,76]
		↑ HT-29 cancer cell proliferation through increased expression of COX-2, VEGF, MMP-9, and PGE2 mediated by epinephrine β_1_- and β_2_-AR signaling	[77]
		↑ HT-29 cancer cell migration and invasion through two signaling pathways (p-Smad3/Snail and HIF-1α/Snail), both of which are activated by NE-mediated increased secretion of TGF-β1	[78]
		↓ tumor progression and metastasis in colorectal cancer patients	[48]
	Parasympathetic	↑ Caco-2 and HCT-8 cancer cell proliferation and inhibition of apoptosis mediated by α7nAChR signaling	[79,80]
		↑ cancer progression and metastasis through α9nAChR signaling in colorectal cancer patients	[4,48]
	Sensory	↑ tumor growth in colorectal cancer patients through RAMP1 signaling mediated by CGRP	[4,81]
**Pancreatic** **Cancer**	Sympathetic	↑ PNI development and progression in MIA PaCa-2 and BxPC-3 cells through increased NGF, MMP-2, and MMP-9 expression induced by STAT3 transcription factor activation mediated by NE-β-AR-PKA signaling	[82]
		↑ neoaxonogenesis and cancer progression in mice with KPC-induced tumors through exosomal packed RNA molecules with internal m^6^A, which were induced by downregulating ALKBH5 in tumor cells after NE signaling	[83]
		↑ neoaxonogenesis in KPC mice and patients through β_2_-AR signaling-mediated NGF secretion from cancer cells, and subsequent stimulation of tumor growth by NE released from newly formed nerves	[84]
		↑ neoangiogenesis and tumor growth in mice with MIA PaCa-2 or BxPC-3-induced tumors via increased expression of VEGF, MMP-2 and MMP-9 induced by transcription factor HIF-1α activation through β_2_-AR signaling	[85]
	Parasympathetic	↑ HPAF, Capan1, and Colo357 cancer cell metastasis and ↑ cancer growth in mice with HPAF-induced tumors through MUC4 overexpression induced by JAK2/STAT3 signaling pathway, activated by α7nAChR	[86]
		↓ tumor growth in KPC mice through M1R-mediated inhibition of MAPK/EGFR and PI3K/AKT pathways, and also suppression of CD11b+ myeloid cells	[87]
		↑ cancer progression and metastasis in pancreatic cancer patients through M3R acetylcholine signaling	[88]
	Sensory	↑ PNI of PANC-1 and MIA PaCa-2 cancer cells during the early stage of tumor formation through SP release and NK-1R signaling, which is stimulated by MMP-1 and PAR1 interaction	[89]
		↑ cancer development and progression in PKCY and PKCT mice	[53]
		↑ cancer progression and metastasis in mice with induced tumors and pancreatic cancer patients through glutamate-mediated activation of the CAMK4-CREB1 signaling pathway, and subsequent increased expression of NMDAR2D receptors on cancer cells at pseudo-synaptic contacts between sensory nerve fibers and cancer cells	[90]
**Head and Neck** **Cancer**	Sympathetic	↑ neoaxonogenesis and an increasing number of intratumoral sympathetic nerve fibers through transdifferentiation of sensory neurons to adrenergic neurons after loss of p53 function and secretion of cancer exosomes containing miRs (miR-21, miR-197, miR-324), and subsequent stimulation of tumor growth by these nerve fibers in mice with PCI-13-induced tumors	[30]
		↑ neoangiogenesis and cancer progression through increased expression of VEGF, MMP-2, MMP-9, and IL-6 in cancer cell lines (SCC9, SCC15, SCC25, HONE-1, HNE-1, CNE-1), and also direct influence of NE on these cancer cells, both mediated by β_1_- and β_2_-AR	[91,92]
	Sensory	↑ neoaxonogenesis and tumor growth in mice with mEERL-induced tumors and patients with head and neck tumors through increased nerve density mediated by secretion of cancer-derived exosomes containing axonal guidance molecule ephrin-B1, which activates EPH receptor-MAPK signaling	[7]
		↑ mEERL, SCC1 and SCC47 cancer cell proliferation and migration through NK-1R signaling, activated by SP	[93]
**Gastric** **Cancer**	Sympathetic	↓ tumor progression and metastasis in gastric cancer patients through β_1_- and β_2_-AR signaling	[94]
		↑ MKN45, NUGC3, and MKN74 cancer cell proliferation and migration, and ↑ progression and metastasis through NE β-AR in patients	[95]
		↑ neoangiogenesis, cancer progression, and metastasis in mice with induced tumors through increased expression of pro-angiogenic factors (VEGF, MMP-2, MMP-7, MMP-9), and activation of the ERK1/2-JNK-MAPK pathway and transcription factors NF-κB, AP-1, CREB, and STAT3 by NE β_2_-AR signaling	[96]
	Parasympathetic	↑ cancer development and progression in mice through an increase in NGF expression and TrkA signaling, which is induced by acetylcholine-M3R signaling	[34]
		↑ cancer development and progression in INS-GAS mice through activation of Wnt signaling by M3R acetylcholine receptors	[13]
		↑ cancer progression in mouse and human gastric organoids through inhibition of the activity of RING E3 ligase PJA2 (inhibition reduces M3R ubiquitination and degradation) and acetylcholine M3R-mediated activation of TGF-β/SMAD3 signaling pathway	[97]
	Enteric	↑ cancer incidence and growth in rats with MNNG-induced tumors	[98]
	Sensory	↑ tumor growth in gastric cancer patients through RAMP1 signaling mediated by CGRP	[81]
**Lung** **Cancer**	Sympathetic	↑ tumor growth and metastasis in mice through EGFR-mediated inactivation of tumor suppressor LKB1 and increased IL-6 expression induced by β_2_-AR signaling	[99]
		↑ cancer progression in lung cancer patients	[100,101]
		↑ A549 cancer cell migration and invasion through two signaling pathways (p-Smad3/Snail and HIF-1α/Snail), both of which are activated by NE-mediated increased secretion of TGF-β1	[78]
	Parasympathetic	↑ cancer progression in lung cancer patients	[100]
	Sensory	↑ tumor growth in mice with induced tumors, glutamate and GABA-mediated synaptic transmission through synapses between nerve endings and cancer cells	[102,103]
**Ovarian** **Cancer**	Sympathetic	↑ neoaxonogenesis in mice with SKOV3ip1- and HeyA8-induced tumors through BDNF-TrkB signaling, which is triggered by increased NE levels and β_3_-AR signaling, and subsequent stimulation of growth and metastasis by these nerves	[33]
		↑ tumor growth and metastasis through activation of Src protein tyrosine kinase induced by NE-mediated β_2_-AR-cAMP-PKA signaling in SKOV3ip1 cancer cells	[104]
		↑ neoangiogenesis and tumor growth in mice with SKOV3ip1- and HeyA8-induced tumors through increased levels of VEGF, MMP-2, and MMP-9 mediated by β_2_-AR signaling	[105]
**Liver** **Cancer**	Sympathetic	↑ tumor growth in liver cancer patients through β_2_-AR signaling	[2]
		↑ cancer development and tumor growth in rats with liver tumors and liver cancer patients through increased expression of IL-6 and TGF-β in Kupffer cells mediated by NE α_1_-AR signaling	[106]
	Parasympathetic	↑ tumor growth in liver cancer patients through α7nAChR, M1R, and M3R signaling	[2]
**Melanoma**	Sympathetic	↑ cancer development and tumor growth in mice with B16-F10-induced tumors	[107]
		↑ A375 cancer cell proliferation and tumor growth	[108]
		↑ cancer progression in mice with B16-F10-induced tumors through activation of HIF-1α transcription factor mediated by D2 dopamine receptor signaling	[109]
		↑ cancer progression through increased levels of VEGF, MMP-2, IL-6, and IL-8 in A375, C8161, 1174MEL, Me18105, and Hs29-4T melanoma cell lines, mediated by epinephrine and NE-induced β_1_- and β_2_-AR signaling	[47,110]
	Sensory	↑ tumor growth in mice with B16-F10-induced tumors through increased expression of chemokines (CCL2, CCL3, CCL5, CXCL1, CXCL2, and CXCL12), which attracts MDSCs creating an immunosuppressive environment	[52]
		↓ neoangiogenesis and cancer progression in mice with B16-F10-induced tumors	[17]
**Esophageal** **Cancer**	Sympathetic	↑ neoaxonogenesis and potential stimulation of tumor growth in esophageal cancer patients	[111]
		↑ HKESC-1 and HKESC-3 cancer cell proliferation through increased expression of COX2, VEGF, VEGFR, EGF, EGFR, cyclin D_1_, cyclin E_2_, CDK-4, and CDK-6 mediated by epinephrine β_1_- and β_2_-AR signaling	[112,113]
	Sensory	↑ neoaxonogenesis and tumor growth in esophageal cancer patients	[111]

↑—stimulatory effects on cancer; ↓—inhibitory effects on cancer. Abbreviations: α7nAChR—α7-nicotinic acetylcholine receptor; α9nAChR—α9-nicotinic acetylcholine receptor; Akt (PKB)—protein kinase B; ALKBH5—RNA demethylase alkB homologue 5; AR—adrenergic receptor; ARG-1—arginase 1; CAMK4—calcium/calmodulin dependent protein kinase 4; cAMP—cyclic adenosine monophosphate; CCL—chemokine ligand; CDK—cyclin-dependent kinase; CGRP—calcitonin gene-related peptide; COA6—cytochrome c oxidase assembly factor 6; COX-2—cyclooxygenase-2; CREB—cAMP-responsive element-binding protein; CXCL—C-X-C motif chemokine ligand; EGF—epidermal growth factor; EGFR—epidermal growth factor receptor; EPH—Ephrin receptor; ERK 1/2—extracellular signal-regulated kinase 1/2; GABA—γ-aminobutyric acid; GSK-3—glycogen synthase kinase-3; HDAC2—histone deacetylase 2; HIF-1α—hypoxia-inducible factor 1α; IFN-β—interferon-β; IFN-γ—interferon-γ; IL—interleukin; JAK2—Janus kinase 2; LKB1—liver kinase B1; M1R—muscarinic acetylcholine receptor type 1; M3R—muscarinic acetylcholine receptor type 3; m6A—N6-methyladenosine modifications; MAPK—mitogen-activated protein kinase; M-CSF—macrophage colony-stimulating factor; MDSCs—myeloid-derived suppressor cells; MIC-1—macrophage inhibitory cytokine-1; miR—microribonucleic acid; MMP—matrix metalloproteinase; MNNG—N-methyl-N′-nitro-N-nitrosoguanidine; MUC-4—mucin-4; NE—norepinephrine; NEPC—neuroendocrine prostate cancer; NF-κB—nuclear factor κB; NGF—nerve growth factor; NK-1R—neurokinin 1 receptor; NMDAR2D—N-methyl-D-aspartate glutamate receptor subunit 2D; NPY—neuropeptide Y; NPYR—neuropeptide Y receptor; PAR1—protease-activated receptor 1; PDX—patient-derived xenografts; PGE2—prostaglandin E2; PI3K—phosphatidylinositol 3-kinase; PJA2—praja ring finger ubiquitin ligase 2; PKA—protein kinase A; PNI—perineural invasion; RAMP1—receptor activity modifying protein 1; RANKL—receptor activator for nuclear factor-κB ligand; Smad3—mothers against decapentaplegic homolog 3; SP—substance P; ssRNA—single-stranded ribonucleic acid; STAT3—signal transducer and activator of transcription 3; TACR1—tachykinin receptor 1; TGF-β—transforming growth factor-β; TLR7—toll-like receptor 7; TrkA—tropomyosin receptor kinase A; TrkB—tropomyosin receptor kinase B; TSP-1—thrombospondin 1; VEGF—vascular endothelial growth factor.

## 3. Tumor Neoaxonogenesis

As mentioned above, tumors based on their location are innervated by sympathetic, parasympathetic, and sensory nerve fibers. Under normal (physiological) conditions, these fibers regulate tissue and organ functions, repair processes, and homeostasis in the human body [22,25]. In connection with cancer, it has been demonstrated that nerve fibers influence several signaling pathways associated with the development, progression, and metastasis of various types of tumors [11,114]. Cancer cells play a significant role in initiating interactions between nerve fibers and the components of the tumor microenvironment. They produce and release neurotrophic factors, axonal guidance molecules, and exosomes that stimulate and direct the growth of new nerve fibers (axons) into tumor tissue, a process known as neoaxonogenesis [6,7,31]. From a clinical perspective, neoaxonogenesis in tumors is considered an important prognostic characteristic because it increases neural influence on tumors, resulting in the release of neurotransmitters from intratumoral nerve endings. These neurotransmitters then bind to receptors on cancer cells and other cells in the tumor microenvironment, where they can stimulate (or, in some cases, inhibit) signaling pathways associated with further tumor growth [1,27,114]. This fact is supported by findings of several studies, which have shown an association between increased nerve density in tumors and worse prognosis in experimental animals and/or cancer patients [2,4,115]. Therefore, innervation density, defined as the number of nerve profiles identified within a specific area, has been considered an important diagnostic and prognostic clinical tool in several cancers [5]. Innervation density, a measurable parameter of cancer neoaxonogenesis, can be detected by immunohistochemistry (IHC) or immunofluorescence (IFC) using antibodies against neuronal protein markers expressed in tumors. The IHC and IFC staining procedures involve the use of antibodies against common neuronal markers, such as protein gene product 9.5 (PGP9.5) and protein S100, as well as antibodies against specific markers of nerve fibers (e.g., tyrosine hydroxylase—TH or neuropeptide Y—NPY for sympathetic nerve fibers detection; vesicular acetylcholine transporter—VAChT for parasympathetic nerve fibers detection; substance P—SP or calcitonin-gene related peptide—CGRP for sensory nerve fibers detection). Except for innervation density, cancer neoaxonogenesis can be evaluated by other methods, such as co-cultivation of cancer cells with neurons (e.g., dorsal root ganglion neurons—DRG, pheochromocytoma cells—PC12) or detection of gene expression and protein levels of factors associated with nerve fiber growth into tumors (e.g., neurotrophic factors, axonal guidance molecules, molecules packed in exosomes) [5,7,8,114].

### 3.1. Neurotrophic Factors

**Figure 3:**
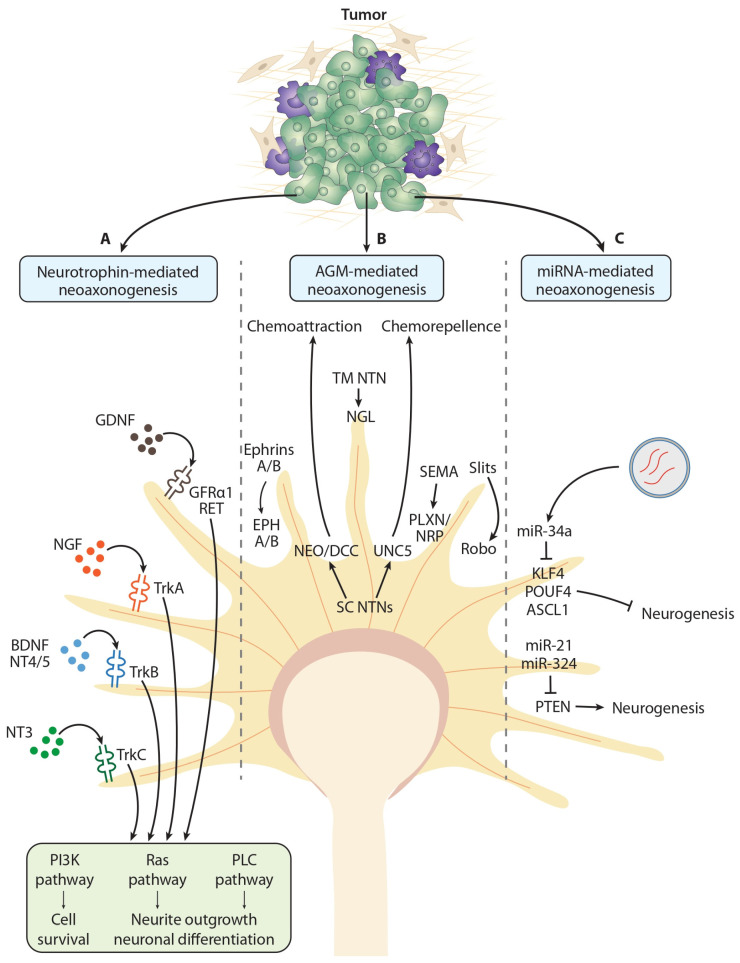
The influence of cancer cells-derived neurotrophic factors, AGM, and exosomes on tumors. Cancer cells initiate interactions between nerve fibers and components of the tumor microenvironment. Through the production and release of (**A**) neurotrophic factors (nerve growth factor—NGF, brain-derived neurotrophic factor—BDNF, glial cell line-derived neurotrophic factor—GDNF, neurotrophin-4/5—NT-4/5, neurotrophin-3—NT-3), (**B**) axonal guidance molecules—AGM (netrins, semaphorins, ephrines, slits), and (**C**) exosomes (containing AGM or microRNA—miR) they stimulate and correct the growth of new nerve fibers (axons) into tumors, a process known as neoaxonogenesis. Additionally, other molecules in the tumor microenvironment may stimulate this process, such as granulocyte colony-stimulating factor (G-CSF) and leukemia inhibitory factor (LIF). These molecules and factors may stimulate the proliferation, migration, and dissemination of cancer cells. (**A**)—the effects of neurotrophic factors on cancer neoaxonogenesis and the cancer cells’ activity are mediated by two types of membrane receptors: the p75 neurotrophin receptor (p75^NTR^) and the neurotrophic tyrosine kinase receptors type 1, 2, and 3 (TrkA, TrkB, and TrkC). The effects of GDNF on cancer neoaxonogenesis and cancer cell activity are mediated by the receptor tyrosine kinase rearranged during transfection (RET), which interacts with GDNF only after interacting with the co-receptor GDNF family receptor α1 (GFRα1). Activation of these receptors for neurotrophic factors initiates several downstream signaling pathways, including the phosphatidylinositol 3-kinase (PI3K), Ras, and phospholipase C (PLC) pathways. (**B**)—AGMs, such as netrins, semaphorins, ephrins, and slits, mediate the chemoattraction or chemorepellence of growing axons during neoaxonogenesis and modulate cancer cell activity. Netrins (NTNs) are classified into two groups: secretory netrins (SC) and membrane-anchored (TM) netrins. SC NTNs affect cancer neoaxonogenesis and the activity of cancer cells through receptors from the neogenin (NEO), deleted in colorectal cancer (DCC), or uncoordinated 5 (UNC5) groups. TM NTNs, on the other hand, interact with transmembrane proteins from the netrin-G ligand (NGL) group. Semaphorins (SEMA) are classified into eight groups and affect cancer neoaxonogenesis and the activity of cancer cells through two types of receptors: plexins (PLXN) and neuropilins (NRP). Ephrins are classified into two types: type A and B ephrins, and both interact with tyrosine kinase receptors for ephrins (EPHA/B) to mediate their effects on cancer neoaxonogenesis and cancer cell activity. Regarding Slits, three Slit homologues (Slit1, Slit2, Slit3) have been identified that act via the transmembrane Roundabout (Robo) receptors. (**C**)—MicroRNAs (miRs) packed in tumor-secreted exosomes have different effects on cancer neoaxonogenesis. For instance, miR-34a inhibits neoaxonogenesis by downregulating the transcription factors Krüppel-like factor 4 (KLF4), POU class 5 homeobox 1 (POU5F1), and achaete-scute homolog 1 (ASCL1). By contrast, miR-21 and miR-324 stimulate neoaxonogenesis by downregulating phosphatase and tensin homolog (PTEN). Nowadays, neoaxonogenesis in tumors is considered an important hallmark because it increases neural influence and the amount of neurotransmitters secreted from intratumoral nerve endings, which predominantly stimulate cancer progression (modified according to Vermeer et al. [26]).

Neurotrophic factors, also known as neurotrophins, represent a group of structurally and functionally related growth factors that play an important role in the development of the nervous system during embryogenesis by supporting the growth of neurites, as well as survival, migration, regeneration, and differentiation of CNS and PNS cells [26,116]. The biological effects of neurotrophic factors are mediated by two types of membrane receptors: the common neurotrophin receptor p75 (p75^NTR^) and neurotrophic tyrosine kinase receptor types 1, 2, and 3 (TrkA, TrkB, TrkC). Regarding cancer, two types of neurotrophic factor-mediated effects have been demonstrated: stimulation of neoaxonogenesis and direct influence on cancer cell activity [26,116,117]. Several studies have shown that nerve growth factor (NGF), brain-derived neurotrophic factor (BDNF), and glial cell line-derived neurotrophic factor (GDNF) are the most important neurotrophic factors that affect tumors (Figure 3) [6,118,119]. Therefore, we will discuss these neurotrophins in more detail in the following sections.

#### 3.1.1. Nerve Growth Factor

Nerve growth factor (NGF) was the first neurotrophic factor to be discovered and described in terms of its functions within the CNS and PNS [119,120,121]. Outside of the nervous system, several studies have demonstrated the presence of abundant levels of NGF and its receptors (TrkA and p75^NTR^) in the tumor tissue using gene expression detection and immunohistochemical staining [115,116]. This has been associated with an increased number of autonomic and sensory nerve fibers, as well as tumor growth and metastasis [6,12,117,122]. Furthermore, the administration of an anti-NGF antibody or a TrkA receptor inhibitor was associated with decreased neurotrophic activity promoting neoaxonogenesis in breast and esophageal cancer cells, as well as the inhibition of tumor growth and metastasis in a mouse model of breast cancer [6,12,117,122]. In a mouse model of gastric cancer, Hayakawa et al. [34] demonstrated that acetylcholine increases NGF expression, which is associated with enteric nerve fiber growth stimulation and cancer cell proliferation. Similarly, increased sympathetic nerve fiber density and β_2_-AR signaling in human gastric tumors and mouse pancreatic tumors led to increased gene expression and release of NGF from cancer cells, which was associated with stimulation of neoaxonogenesis and NE accumulation in tumors, and subsequent stimulation of tumor progression [84,95,119]. As previously mentioned, neurotrophic factors, including NGF, directly influence not only neoaxonogenesis in tumors but also the activity of cancer cells, thereby regulating processes associated with cancer growth and metastasis. In breast cancer specimens, NGF expression has been shown to be associated with the metastatic spread of tumors to lymph nodes. Binding of NGF to TrkA receptors activates mitogen-activated protein kinases (MAPKs), stimulating cancer cell proliferation, growth, and metastasis [6,123]. Similarly, in human oral cavity and salivary gland cancer cells, Alkhadar et al. [124] demonstrated that the binding of NGF to TrkA receptors activates the phosphatidylinositol-3-kinase (PI3K)/protein kinase B (Akt) signaling pathway, which promotes the migration and spread of cancer cells. The administration of MK2206, a selective inhibitor of the PI3K/Akt signaling pathway, alone or in combination with NGF, prevented Akt phosphorylation and consequently cancer cell migration [124]. Furthermore, in melanoma, Yin et al. [32] found that TrkA receptor activation following NGF binding reduces the infiltration of the tumor microenvironment by T lymphocytes and natural killer (NK) cells, leading to inhibition of antitumor immunity. The binding of NGF to p75^NTR^ receptors activates nuclear factor kappa-B (NF-κB), which mediates resistance to apoptosis and stimulates cancer cell proliferation [125]. Finally, the release of NGF from cancer cells can increase the expression and release of proangiogenic molecules (e.g, vascular endothelial growth factor—VEGF), which stimulate the growth, migration, invasion, tubular formation, and monolayer permeability of endothelial cells, thus promoting neoangiogenesis in tumors [126,127].

In addition to the mature form of NGF, the NGF precursor (proNGF), which is primarily responsible for inducing neuronal apoptosis in the nervous system, also has a significant effect on tumors [128]. In prostate cancer specimens, elevated levels of proNGF in the cytoplasm of cancer cells have been observed to be associated with a higher Gleason score. Although the authors did not test innervation density in tumor specimens, their co-culture experiments with PC-3 prostate cancer cells and neuronal cells revealed that proNGF produced by cancer cells stimulates neoaxonogenesis [129,130]. Similarly, increased levels of proNGF were demonstrated in the thyroid tumor tissues compared to normal thyroid tissue [131]. Additionally, proNGF stimulates the metastatic spread of melanoma and breast cancer cells to lymph nodes by binding to TrkA receptors [132,133].

#### 3.1.2. Brain-Derived Neurotrophic Factor

Brain-derived neurotrophic factor (BDNF) is another important protein from the neurotrophin group that is responsible for supporting the growth, functioning, and plasticity of neuronal cells within the nervous system. However, elevated levels of BDNF have also been demonstrated in the tissues of several types of tumors, and binding BDNF to TrkB or p75^NTR^ receptors is associated with the activation of signaling pathways that directly influence cancer cell activity and tumor neoaxonogenesis [134,135]. In a mouse model of ovarian cancer, Allen et al. [33] demonstrated that increased stress-induced release of NE in tumors leads to increased production and release of BDNF by cancer cells through β_3_-AR signaling. BDNF then binds to the TrkB receptor and induces nerve fiber growth within tumor tissue. Furthermore, a greater number of nerve fibers was significantly associated with higher levels of NE and BDNF in tumors, as well as with the overall survival of ovarian cancer patients [33]. Increased gene expression and protein levels of BDNF and TrkB receptors, as well as decreased E-cadherin expression, have been demonstrated in human head and neck cancer tissue samples. This has been associated with the stimulation of cancer cell migration and metastasis through the stimulation of epithelial-mesenchymal transition (EMT), as well as a poorer prognosis in patients [134,136]. Similarly, increased expression of BDNF and TrkB receptors has been demonstrated in melanoma, and this was associated with tumor stage, histological subtype, and reduced patient survival [137]. In breast cancer, BDNF together with neurotrophin-4/5 (NT4/5) promotes cancer cell survival and resistance to apoptosis by simultaneous activation of p75^NTR^ and TrkB receptors [138]. Additionally, BDNF stimulates tumor cell proliferation, metastasis, and neoaxonogenesis in this type of tumor, and elevated BDNF levels are associated with poorer clinical outcomes and reduced survival in cancer patients [114,139,140]. In colorectal cancer, Huang et al. [141] demonstrated the stimulatory effect of BDNF on cancer cell migration via increased expression of heme oxygenase-1 (HO-1) and VEGF. Furthermore, Renz et al. [84] demonstrated that 35% of samples obtained from patients with pancreatic stromal tumors expressed BDNF, which was positively correlated with the presence of a larger number of nerve fibers in tumors.

#### 3.1.3. Glial Cell Line-Derived Neurotrophic Factor

There is currently growing evidence of the influence of glial cell line-derived neurotrophic factor (GDNF) on nerve fiber growth and cancer cell activity. In this context, Ban et al. [142] demonstrated the stimulatory effect of GDNF on prostate cancer cell proliferation and perineural invasion (PNI) by activating the rearranged during transfection (RET) receptor tyrosine kinase signaling pathway. High levels of GDNF expression have been observed in pancreatic cancer cells and have been associated with cancer cell migration and PNI through activation of the PI3K and MAPK signaling pathways. They have also been associated with increased intensity of cancer-induced pain [114,143]. Additionally, increased expression of the CD74 protein characteristic of antigen-presenting cells in the pancreatic tumors stimulates GDNF secretion, which promotes nerve fiber growth [144]. In colorectal cancer, Huang et al. [145] found that GDNF stimulates cancer cell migration by increasing the interaction between VEGF and its receptor, which is regulated by the p38, PI3K/Akt, and hypoxia-inducible factor 1-α (HIF-1α) signaling pathways.

### 3.2. Axonal Guidance Molecules

Axonal guidance molecules (AGMs) are key regulatory factors in the development of neural circuits within the nervous system. They are responsible for the chemoattraction or chemorepellence of growing axons, correcting their growth direction until they reach target areas [10,146]. Several studies suggest that AGMs, like neurotrophic factors, can stimulate the process of neoaxonogenesis in tumors and the activity of cancer cells, thereby promoting cancer progression [10,26]. In contrast, several AGMs were also associated with antitumor effects, including reduced cancer cell migration and metastasis, reduced angiogenesis, and reduced tumor growth. To clarify these dual effects of AGMs, more research is needed to investigate the role of various AGMs in different tumor types. In the context of the AGM-mediated effects on peripheral tumors, the most important groups of AGMs are netrins, semaphorins, ephrins, and slits (Figure 3) [40,146].

#### 3.2.1. Netrins

Netrins (NTNs) are a group of extracellular proteins belonging to the AGM family that play important roles in cell migration and the correction of axonal growth direction during nervous system development [146,147]. Six types of NTN have been identified in mammals, which are divided into two groups: secretory NTN (NTN-1, NTN-3, NTN-4, and NTN-5) and membrane-anchored NTN by glycosylphosphatidylinositol—GPI (NTN-G1 and NTN-G2). Secretory NTNs mediate the chemoattraction of growing axons via receptors from the neogenin (NEO) and deleted in colorectal cancer (DCC) group, while the chemorepellence of axons is mediated by receptors from the uncoordinated 5 homologs (UNC5) group. Membrane-anchored NTNs do not bind to secretory NTN receptors but instead interact with transmembrane proteins from the netrin-G ligand (NGL-1 and NGL-2) group [147,148,149]. The functions of NTNs are not limited to the development of the nervous system, but they can also affect cancer progression [40,150]. A study by Haidar et al. [151] showed that NTN-1, released from cancer cells in a mouse model of pancreatic intraepithelial neoplasia (PanIN), the precursor lesions of pancreatic cancer, stimulates sympathetic axonal growth by binding to the DCC receptor. This process was associated with increased proliferation of precancerous cells and PanIN progression. Furthermore, NTN-1 expression was confirmed in human pancreatic cancer precursor lesions [151]. Using a rat model of bone pain induced by breast cancer, Gong et al. [152] identified an increase in the number of nociceptive sensory nerve fibers and increased expression of NTN-1 and its DCC receptor at sites of metastatic bone lesions. Increased expression of NTN-1 has been demonstrated in breast cancer cell lines and in metastatic breast cancer specimens from patients. Experimentally induced decreases in NTN-1 expression led to stimulation of cancer cell apoptosis and inhibition of metastasis [153]. Regarding colorectal cancer, Li et al. [150] discovered that serum concentrations of NTN-1 were higher in cancer patients than in healthy individuals. Furthermore, their results showed that higher levels of NTN-1 are associated with an increased risk of developing colorectal cancer [150]. Elevated levels of NTN-G1, detected in liver cancer cell lines and liver tumor tissue samples, have been associated with increased cancer cell proliferation and migration, EMT, reduced cancer cell apoptosis, stimulation of tumor progression, and poorer overall survival rates [147]. Similarly, Zhao et al. [154] found that increased NTN-1 expression in lung tumors was associated with a worse prognosis. Additionally, the transfection of human A549 lung cancer cells with NTN-1 small interfering RNAs (siRNAs) effectively knocked down NTN-1 and increased the rate of chemotherapy-induced apoptosis [154]. Using human and mouse breast cancer cells, as well as a mouse model of breast cancer, Larrieu-Lahargue et al. [155] showed that increased NTN-4 induces neolymphangiogenesis and neoangiogenesis, thereby supporting the metastatic process through activating small guanosine triphosphatase (GTPase) and Src family kinases/Focal adhesion kinase (FAK), and downregulating tight junction proteins. In contrast, several studies demonstrated the antitumor effects of NTN-4. For example, Reuten et al. [156] demonstrated in several cancer types, including melanoma, breast cancer, and pancreatic cancer, that the presence of NTN-4 in the basement membrane (BM), which is produced by tumor-associated fibroblasts and endothelial cells, reduces BM stiffness and prevents cancer cell migration and metastasis. Similar antitumor effects of NTN-4 have been demonstrated in other breast cancer studies. Here, the authors revealed an association between NTN-4 expression in cancer cells and tumor specimens and the inhibition of cancer cell migration and increased patient survival (Table 2; Figure 3) [157,158].

#### 3.2.2. Semaphorins

Semaphorins (SEMA) represent a large group of secretory and membrane-bound glycoproteins that play an important role in guiding the direction of axonal growth cone growth during nervous system development [146,159]. Based on structural and sequence homology, SEMAs are classified into eight classes (SEMA1-7 and SEMA V), five of which (SEMA3-7) have been identified in vertebrates. Third-class SEMAs (SEMA3A-G) are secretory proteins, fourth- to sixth-class SEMAs (SEMA4A-G, SEMA5A-B, and SEMA6A-D) are transmembrane proteins, and seventh-class SEMAs (SEMA7A) are anchored in the cell membrane by GPI. In the body, SEMAs act on two types of receptors that mediate their biological effects: plexins (PLXN A1-A4; PLXN B1-B3; PLXN C1; PLXN D1) and neuropilins (NRP1; NRP2) [35,40,159]. The stimulating effect of SEMA molecules on cancer cell activity and nerve fiber growth in tumors is well-documented. For example, Jurcak et al. [160] demonstrated, through co-culture and in vivo experiments, that pancreatic cancer cells promote their own migration, metastasis, and tumor tissue innervation by secreting SEMA3D, which binds to the PLXN D1 receptor. Elevated levels of SEMA3D and SEMA3A, as well as their receptors PLXN D1 (SEMA3D), PLXN A1-A4, and NRP (SEMA3A), were positively correlated with the presence of PNI and poor clinical outcomes in human pancreatic cancer samples [160,161]. Hung et al. [35] demonstrated that increased SEMA3A expression in pancreatic cancer cells and tumors induced in mice stimulates migration and spread of cancer cells, as well as tumor neoaxonogenesis and growth by binding to PLXN A1 and NRP2 receptors and subsequently activating the MAPK. Experimental co-cultivation of prostate cancer cell lines with dorsal root ganglion (DRG) neurons revealed increased SEMA4F expression in cancer cells, associated with an increased number of growing nerve fibers and their average length [8]. Ding et al. [162] demonstrated that transfecting prostate cancer cells with the SEMA4F retrovirus increased their proliferation and migration rates. They also reported higher levels of SEMA4F in prostate tumors than in healthy prostate epithelium, which were positively correlated with innervation density in tumors. Finally, patients with prostate tumors that exhibited high levels of SEMA4F also had a significantly higher risk of tumor recurrence [162]. Elevated levels of SEMA4C and SEMA4D were associated with the stimulation of cancer cell proliferation, tumor growth, and metastasis in breast cancer cell lines and mouse tumor tissues [163,164]. Similarly, increased levels of SEMA7A have been found in tumors of postpartum patients. Using breast cancer cell lines and a postpartum breast cancer mouse model, Tarullo et al. [165] demonstrated that SEMA7A promotes cancer progression in postpartum patients through increased expression of cyclooxygenase-2 (COX-2) and collagen deposition mediated by fibroblasts in the tumor microenvironment. Third-class SEMAs have stimulatory effects on pancreatic cancer progression but appear to inhibit breast cancer, colorectal cancer, and melanoma growth. For example, Rolny et al. [166] demonstrated in an experimental breast cancer model that increased SEMA3B expression inhibits tumor growth but induces metastatic spread by increasing interleukin-8 (IL-8) production in cancer cells, which attracts tumor-associated macrophages. Casazza et al. [167] demonstrated that SEMA3A expression in mouse breast tumors was associated with inhibition of tumor growth, neoangiogenesis, and metastasis. Finally, co-cultivation of colorectal and melanoma cancer cells with DRG neurons increased the expression of third-class SEMAs, which inhibited axonal growth via NRP1 receptors (Table 2; Figure 3) [168].

#### 3.2.3. Ephrins

Ephrins are membrane-bound ligands that belong to the AGM group. They are divided into two subgroups based on their structure and method of attachment to the plasma membrane: type A ephrins, anchored by GPI, and type B ephrins, which contain a transmembrane domain with a short terminal cytoplasmic domain [146,169]. Five different type A ephrin molecules (ephrin-A1–A5) have been identified in humans, interacting with nine types of tyrosine kinase receptors for ephrin-A (EPHA1-8; EPHA10), as well as three different type B ephrin molecules (ephrin-B1–B3), interacting with five types of tyrosine kinase receptors for ephrin-B (EPHB1-4; EPHB6) [40,146]. Experiments conducted by Madeo et al. [7] revealed that stimulating the rat pheochromocytoma cell line (PC12) with head and neck cancer-derived exosomes containing ephrin-B1 promotes tumor tissue innervation by activating MAPKs, which are responsible for nerve fiber growth. Using human colorectal cancer cell lines and in patients with colorectal cancer, Yamamoto et al. [170] found that reduced expression of ephrin-A1 was associated with decreased cancer cell proliferative and migratory activity, lower risk of tumor recurrence, and an increased survival of cancer patients. However, increased expression of ephrin-B2 in a mouse model of colorectal cancer stimulated neoangiogenesis, but surprisingly, reduced tumor growth due to structural abnormalities in the new blood vessels [171]. Notably, colorectal cancer tissue samples demonstrated higher ephrin-B2 expression than surrounding healthy tissue [172]. Increased expression of the EPHA2 and EPHA10 receptors and decreased expression of the EPHA4 receptors were demonstrated in breast tumor samples [163,173,174]. And finally, decreased expression of ephrin-A1 in human and mouse breast tumors has been associated with EPHA2-mediated tumor growth stimulation and a poorer cancer prognosis (Table 2; Figure 3) [175].

#### 3.2.4. Slits

Slit proteins are secretory glycoproteins of the extracellular matrix that act as chemorepellents during nervous system development by preventing axons from crossing the midline. Three Slit homologs (Slit1, Slit2, Slit3) have been identified, which act in the organism via four types of transmembrane Roundabout (Robo) receptors (Robo1-4) [40,146,159]. In pancreatic cancer, Biankin et al. [176] revealed mutagenic changes in genes of signaling pathways activated by Slit-Robo interactions, supporting the potential involvement of AGM genes in pancreatic cancer carcinogenesis. Similarly, in pancreatic cancer, Secq et al. [177] demonstrated that the Slit2 protein, which is secreted by tumor microenvironment fibroblasts, can promote the growth of DRG neuron nerve fibers, proliferative and migratory activity of Schwann cells, and thus neural remodeling in pancreatic cancer. Reduced Slit2 expression has been demonstrated in human colorectal and gastric cancer cell lines, as well as patient tumor tissues, compared to the surrounding healthy tissue. The binding of Slit2 to Robo1 receptors leads to the inhibition of cancer cell migration and EMT, which is mediated via ubiquitin-specific protease 33 (USP33) [178,179]. On the other hand, Yao et al. [180] found elevated serum levels of Slit2 in patients with colorectal cancer compared to healthy individuals. Blocking the interaction between Slit2 and Robo1 receptors inhibited cancer cell migration and metastasis. In breast cancer, increased Slit2 expression has been shown to inhibit cancer cell migration by inactivating the β-catenin and PI3K/Akt signaling pathways or modulating cell adhesion [181]. In contrast, a study conducted by Padmanaban et al. [66] found that Slit2 promotes breast cancer growth and metastasis by stimulating the release of the neuropeptide substance P (Figure 3).

**Table 2:** An overview of the effects of axonal guidance molecules on cancer.

Group of AGMs	Type of Protein	Cancer Type	Effects on Cancer	Studies
**Netrins**	NTN-1	Pancreatic cancer	↑ sympathetic neoaxonogenesis and its association with proliferation of precancerous cells and PanIN progression in mice through DCC receptor signaling	[151]
		Breast cancer	Mediating cancer-induced bone pain through DCC receptor signaling activated increase in the number of sensory nerve fibers in a rat model ↓ cancer cell apoptosis and ↑ of metastasis into the lungs in mouse models	[152,153]
		Colorectal cancer	An association between higher serum concentrations and an increased risk of developing CRC	[150]
		Lung cancer	An association between increased expression in lung tumor specimens and worse patient prognosis	[154]
	NTN-4	Breast cancer	↑ neolymphangiogenesis, neoangiogenesis and metastasis, by activating small GTPases and Src/FAK kinases, and by downregulating tight junction proteins ↓ cancer cell migration and metastasis through reduction of BM stiffness An association between increased expression in cancer cells and tumor specimens and ↓ cancer cell migration and increased patient survival	[155,156,157,158]
		Pancreatic cancer	↓ cancer cell migration and metastasis through reduction of BM stiffness	[156]
		Melanoma	↓ cancer cell migration and metastasis through reduction of BM stiffness	[156]
	NTN-G1	Liver cancer	An association between elevated levels in liver cancer cell lines and tumor specimens and ↑ cancer cell proliferation and migration, EMT, ↓ cancer cell apoptosis, ↑ tumor progression, and poorer overall survival rates	[147]
**Semaphorins**	SEMA3A-G	Melanoma	↓ neoaxonogenesis in DRG neurons by co-cultivation with cancer cells mediated through NRP1 receptors	[168]
		Colorectal cancer	↓ neoaxonogenesis in DRG neurons by co-cultivation with cancer cells mediated through NRP1 receptors	[168]
	SEMA3A	Pancreatic cancer	A correlation between elevated levels and the presence of PNI, as well as poor clinical outcomes ↑ cancer cell migration and spread, as well as neoaxonogenesis in mouse tumors through PLXN A1/NRP2-MAPK signaling	[35,161]
		Breast cancer	↓ tumor growth, neoangiogenesis, and metastasis in mice	[167]
	SEMA3B	Breast cancer	↓ tumor growth in mice, and ↑ metastatic spread by increasing IL-8 production in cancer cells, which attracts tumor-associated macrophages	[167]
	SEMA3D	Pancreatic cancer	↑ migration and metastasis of cancer cells, and neoaxonogenesis in a mouse pancreatic cancer model through PLXN D1 receptors A correlation between elevated levels and the presence of PNI, as well as poor clinical outcomes	[160]
	SEMA4C	Breast cancer	↑ cancer cell proliferation, tumor growth, and metastasis	[163]
	SEMA4D	Breast cancer	↑ cancer cell proliferation, tumor growth, and metastasis	[164]
	SEMA4F	Prostate cancer	↑ neoaxonogenesis in DRG neurons through co-cultivation with cancer cells ↑ human cancer cell proliferation and migration A correlation between higher levels and innervation density in human tumor samples, as well as higher risk of cancer recurrence	[8,162]
	SEMA7B	Breast cancer	↑ cancer progression through increased COX-2 expression and fibroblast-mediated collagen deposition	[165]
**Ephrins**	Ephrin-A1	Colorectal cancer	↑ cancer cell proliferation and migration An association between increased expression and lower risk of tumor recurrence, as well as decreased patient survival	[170]
		Breast cancer	↑ tumor growth in mice through EPHA2 signaling An association between increased expression and poor prognosis	[175]
	Ephrin-B1	Head and neck cancer	↑ neoaxonogenesis in PC12 cells after stimulation by cancer-derived exosomes through ephrin-B1-EPH-MAPK signaling	[7]
	Ephrin-B2	Colorectal cancer	↑ neoangiogenesis and ↓ tumor growth in mice	[171]
**Slits**	Slit2	Pancreatic cancer	↑ neoaxonogenesis in DRG neurons, as well as proliferation and migration of Schwann cells after cultivation with tumor microenvironment media	[177]
		Gastric cancer	↓ cancer cell migration and EMT via Robo1-USP33 pathway	[179]
		Colorectal cancer	↓ cancer cell migration and EMT via Robo1-USP33 pathway ↑ cancer cell migration and metastasis in mice	[178,180]
		Breast cancer	↓ cancer cell migration by inactivating the β-catenin and PI3K/Akt signaling pathways or modulating cell adhesion ↑ tumor growth and metastasis via the release of SP in mice	[66,181]

↑—stimulatory effects; ↓—inhibitory effects. Abbreviations: AGMs—axonal guidance molecules; BM—basement membrane; COX-2—cyclooxygenase-2; CRC—colorectal cancer; DCC—deleted in colorectal cancer receptor; DRG—dorsal root ganglion; EMT—epithelial-mesenchymal transition; EPH—tyrosine kinase receptors for ephrins; FAK—focal adhesion kinase; GTPase—guanosine triphosphatase; IL—interleukin; MAPK—mitogen-activated protein kinase; NRP—neuropilins; NTNs—netrins; PanIN—pancreatic intraepithelial neoplasia; PC12—rat pheochromocytoma cells; PI3K—phosphatidylinositol 3-kinase; PLXN—plexins; PNI—perineural invasion; Robo—roundabout receptors; SEMA—semaphorins; SP—substance P; USP33—ubiquitin-specific protease 33.

### 3.3. Exosomes

In addition to neurotrophic factors and AGMs, another important mechanism that activates the process of neoaxonogenesis in tumors is the secretion and mutual exchange of small endocytic vesicles (exosomes) between neighboring or distant cancer cells. Exosomes represent a small population of extracellular vesicles ranging in size from 30 to 150 nm [7,182,183]. In addition to their transport function, exosomes protect against adverse extracellular conditions, such as the low pH of the tumor microenvironment [182]. Exosomes are formed through endocytosis and contain (transport) several important functional molecules, including DNA, RNA (mRNA and miRNA), lipids, and proteins. The composition of exosomes is largely influenced by the type of cell and its physiological state. For instance, tumor exosomes differ in composition from those of non-tumoral cells [26,184].

A recently published study conducted by Chen et al. [83] demonstrated in mouse model of pancreatic cancer that stress-induced activation of sympathetic nerve fibers, which leads to the NE release, decreases the levels of the RNA demethylase AlkB homolog 5 (ALKBH5) in cancer cells. The deficiency of ALKBH5 in pancreatic cancer cells causes an increased incidence of N6-methyladenosine (m6A) modification in RNA molecules. This modified RNA is incorporated into the exosomes produced by cancer cells and transported to nerve fibers in the tumor microenvironment, where it stimulates neoaxonogenesis and pancreatic cancer progression [83].

#### 3.3.1. Exosome-Packed AGM 

Several studies have shown that various types of cells, including cancer cells, produce and release exosomes containing AGMs, which influence nerve fiber ingrowth. In this context, Gong et al. [185] demonstrated that several types of cells release exosomes containing ephrin-B2, and that the addition of these exosomes to cells induces reverse signaling of ephrin-B1 and chemorepellence of axons. Similarly, Lopez-Verrilli et al. [186] found that dedifferentiated Schwann cells secrete exosomes containing p75^NTR^ receptors that enter axons via endocytosis. There, they promote axon regeneration by inhibiting the activity of the small GTPase RhoA. Stimulating the PC12 cell line with exosomes containing ephrin-B1, obtained from mouse and human head and neck tumors, induced nerve fiber growth by binding ephrin-B1 to the EPH tyrosine kinase receptor, which subsequently activates the MAPK signaling pathway [7]. Similarly, Lucido et al. [187] demonstrated that exosomes obtained from cervical cancer cells stimulate nerve fiber growth in PC12 cells (Figure 3).

#### 3.3.2. Exosome-Packed MicroRNAs

MicroRNAs (miRNAs or miRs) represent a subclass of small single-stranded non-coding RNAs typically comprising 18–25 nucleotides that primarily regulate and inhibit gene expression at the post-transcriptional level. MicroRNAs influence the expression of up to 60% of human genes, thereby playing a role in critical cellular processes such as proliferation, differentiation, and apoptosis, all of which are often dysregulated during oncogenesis [188]. Therefore, genomic aberrations, epigenetic changes, or mutations of miRNAs that affect their processing or activity may nurture a microenvironment conducive to cancer initiation [114]. Accumulating evidence indicates that tumor-secreted exosomes transport functional miRNAs that can induce neural reprogramming, a process frequently observed in developing malignancies. These findings imply that exosome-packed miRNAs function as important mediators of pathological neoaxonogenesis in the tumor microenvironment [30,114,189]. A study conducted by Restaino et al. [93] revealed distinct innervation patterns in human papillomavirus-positive (HPV+) and HPV-negative (HPV-) head and neck cancers, as well as in high-grade ovarian carcinomas. A detailed analysis of cancer-derived exosomes revealed the presence of miR-1972, whose expression levels correlate with a reduced patient survival rate and may mediate cancer innervation through neuronal regulation [93]. Using mouse models of head and neck cancer, Amit et al. [30] conducted a study that revealed elevated levels of miR-34a in exosomes purified from p53-sufficient cells, which have been demonstrated to suppress neuritogenesis by inhibiting axonal growth. Conversely, loss of p53 resulted in significantly decreased levels of miR-34a and an accumulation of proneuritogenic miRs, particularly miR-21 and miR-324-5p. Additionally, inhibiting miR-21, miR-197, and miR-324 decreased nerve outgrowth in cultured trigeminal ganglion neurons with tumor exosomes derived from tumors lacking miR-34a and p53 [30]. Several studies have investigated the association between neoaxonogenesis and PNI regulated by exosome-packed miRs. Liu et al. [190] found that the expression levels of miR-429 were markedly decreased in the MIA PaCa-2 and BxPC-3 pancreatic cancer cell lines and in patient pancreatic cancer specimens with PNI compared to non-PNI tumor samples. They also demonstrated increased neurotrophin-3 (NT3) expression in pancreatic cancer cells and tissue specimens with PNI [190]. Restoring miR-429 expression in pancreatic cancer cells suppressed viability and invasion, likely by inhibiting NT3 expression and secretion. Additionally, in co-culture experiments with PC12 cells, they found that cancer-cell conditioned medium treatment significantly increased neurite outgrowth in PC12 cells via NT3-TrkC signaling, which was suppressed by culturing with conditioned media containing miR-429 [190]. These findings are consistent with the findings presented by Song et al. [191], who demonstrated that low miR-429 expression in pancreatic cancer tissue specimens predicts shorter patient survival. Finally, Sim et al. [192] demonstrated that upregulation of miR-370 promotes breast cancer progression and PNI, which correlates with a poor prognosis. In the context of PNI, Li et al. [193] demonstrated that miR-130b, derived from cancer cell exosomes, stimulates the activity and reprogramming of Schwann cells, which was subsequently associated with PNI progression in salivary gland adenoid cystic carcinoma. Apparently, miRs substantially influence tumor neoaxonogenesis by modulating neuronal differentiation, axonal guidance, and PNI. This contributes to cancer progression and adverse clinical outcomes. Their dysregulation in malignancies highlights their potential as prognostic biomarkers and therapeutic targets. Further research is needed to fully elucidate their mechanisms and explore their potential to disrupt tumor innervation and improve patient survival (Figure 3).

### 3.4. Other Molecules of Cancer Microenvironment Involved in Cancer Neoaxonogenesis

Several studies have demonstrated that other molecules in the tumor microenvironment, such as granulocyte colony-stimulating factor (G-CSF) and leukemia inhibitory factor (LIF), significantly influence the stimulation of neoaxonogenesis in tumors. In prostate cancer, administering G-CSF to transgenic mice that express the c-Myc proto-oncogene has been shown to increase the number of parasympathetic nerve fibers in the tumor, which subsequently stimulated tumor growth and metastasis [194]. A clinical study of patients with pancreatic cancer showed that administering G-CSF before chemotherapy reduced hematological adverse effects and subsequently allowed administration of increased doses and duration of chemotherapy, resulting in longer patient survival [195]. On the other hand, administering G-CSF to patients with pancreatic cancer who were simultaneously receiving neoadjuvant therapy before undergoing surgery to remove the tumor was associated with poorer clinical outcomes and reduced patient survival [196]. The overexpression of LIF in pancreatic cancer has been associated with the differentiation and migration of Schwann cells, as well as the growth of nerve fibers in DRG neurons [197].

## 4. Clinical Implications and Limitations

The innervation of cancer by peripheral nerve fibers establishes a direct connection between the brain and tumors. This connection mediates the negative effects of stress response mediators, such as epinephrine, NE, and cortisol, on processes associated with the development, progression, and metastatic spread of tumors, as well as significantly reduces the effectiveness of antitumor therapy [9,50]. Cancer patients especially experience extremely high levels of stress due to painful invasive diagnostic tests, informing of the diagnosis, an uncertain prognosis, the side effects of anticancer therapy, the threat to their lives, and fear of death. From a clinical perspective, it is extremely important to understand the mechanisms by which nerve fibers influence tumors and the processes associated with nerve fiber growth into tumors, both for increasing the effectiveness of antitumor therapy but also as a part of preventive measures aimed at protecting against the potential development of cancer in the future [22,26,163]. Several experimental and clinical studies have described possibilities for reducing the impact of nerve fibers on tumors and thus inhibiting cancer progression. These approaches have the potential to serve as complementary or negative stress effects reducing therapies in cancer patients. The most significant approaches targeting the effects of nerve fibers on tumors include surgical or chemical removal of nerve fibers (denervation), administration of antagonists of receptors for neurotransmitters released from nerve fiber endings (e.g., antagonists of β-adrenergic receptors, muscarinic acetylcholine receptors, NK-1R receptors for substance P), administration of local anesthetics, administration of antibodies against neurotrophic factors, psychotherapy, and the heart rate variability biofeedback (HRV-B) (Table 3) [16,22,59]. However, it should be noted that the effect of nerve fibers on tumors is extremely complex and does not stimulate growth in all types of cancer. In some types of neoplasms, nerve fibers have been shown to exert an antitumor effect, with their diminished influence being associated with stimulation of tumor growth [17,48]. Therefore, further research is needed to evaluate their effectiveness and clarify all associated complications prior to their application in clinical practice. Limiting the stimulatory effects of nerve fibers on tumors, while preserving normal surrounding nerve fiber and tissue functions, requires a thorough assessment of potential neurological side effects and the design of approaches that allow for direct application of the therapeutic agent to the tumor site. Finally, the antitumor effects of substances that limit nerve influence on tumors vary considerably among different cancer types and among patients with the same type of cancer, which significantly limits their potential for widespread use in clinical practice [6,26,64]. This chapter will discuss the most significant approaches targeting nerve–tumor interactions, including the limitations of their translation in clinical practice.

### 4.1. Surgical and Chemical Denervation

Methods of tumor denervation are based on limiting the influence of nerve fibers on tumors by surgical interruption of the nerve course (surgical denervation) or by applying a chemical substance that destroys nerve endings or inhibits the release of neuromediators (chemical denervation) (Table 3) [1,22,198]. For example, in rat fibrosarcoma induced by the administration of BP6-TU2 cells, Lackovicova et al. [199] demonstrated that the chemical ablation of sympathetic nerves through the intraperitoneal administration of 6-hydroxydopamine (6-OHDA) significantly reduced tumor incidence and increased the survival of tumor-bearing animals compared to those that did not undergo sympathetic denervation. Similarly, in a mouse model of prostate cancer, Magnon et al. [1] found that intraperitoneal administration of 6-OHDA, as well as surgical removal of sympathetic nerves by interrupting the hypogastric nerves, prevented prostate cancer development. These mice showed no detectable metastases due to impaired tumor development, compared to control animals, which developed metastases 11 weeks after the application of cancer cells [1]. A study published by Coarfa et al. [198] showed that bilateral denervation, induced by the intraprostatic botulinum toxin application or pelvic ganglion removal, significantly reduced the incidence of prostate tumors and their size in mice. Local denervation of human prostate tumors using botulinum toxin induced cancer cell apoptosis [198]. In rat model of breast cancer, surgical denervation has been shown to inhibit progression and reduce tumor volume [61]. In pancreatic cancer, removing sensory nerves inhibited tumor development and progression in mice [53]. Similarly, in gastric cancer, Zhao et al. [13] demonstrated that surgical denervation (performed via subdiaphragmatic bilateral truncal vagotomy) and chemical denervation (performed via subserosal injection of botulinum toxin along the greater curvature of the anterior side of the stomach) inhibited tumor progression and increased the effectiveness of chemotherapy in mice. In the clinical part of this study, it was demonstrated that vagotomy performed in patients after distal gastrectomy led to a significant reduction in tumor incidence compared to patients without vagotomy, who developed gastric tumors in the anterior and posterior gastric wall [13]. Finally, Ansiaux et al. [200] demonstrated that local injection of botulinum toxin into mouse fibrosarcoma and liver tumor tissues increases tumor oxygenation and perfusion, which leads to an improvement in the tumor response to radiotherapy and chemotherapy. A particularly interesting finding regarding the impact of denervation on a rat model of breast cancer was published by Mitsou et al. [201]. In this study, the authors investigated the effect of radical and persistent denervation on metastatic tumor growth performed by microsurgical in situ approach. The principle of this approach is based on completely denervating the tumor tissue and its surroundings (nerve fibers and blood vessels) and then reconnecting the tumor tissue to the host. This technique effectively delays or impedes the return of innervation for a significant period, creating a critical therapeutic window. Complete denervation has led to the regression of the primary tumors, which has been associated with a strong antitumor effect and prolonged survival. Interestingly, they found that regression was demonstrated not only in denervated tumors, but also in nondenervated tumors (abscopal effect) [201]. Numerous studies have demonstrated that tumor incidence and progression decrease after nerve fiber removal. However, there are also cases in which nerve fiber removal has led to worse clinical outcomes. For instance, Prazeres et al. [17] conducted a study that demonstrated cancer cell apoptosis inhibition, increased proliferating cell numbers, and increased intratumoral blood vessel numbers in mouse melanoma following the chemical denervation of sensory nerves. Similarly, Tatsuta et al. [202] demonstrated an increased incidence of tumors in the anterior and posterior stomach walls of rats in a chemical-induced gastric cancer model after unilateral (anterior, not posterior) and bilateral vagotomy. In addition, the stimulatory effects of surgical denervation have also been demonstrated in breast cancer, where unilateral vagotomy (performed via a cross-sectional cut of the vagus nerve in the mid-neck region) stimulates metastasis into the lungs [18].

Based on the results of the aforementioned studies, surgical or chemical denervation approaches show promising therapeutic potential in inhibiting tumor progression. However, these approaches have several limitations, making their application to patients problematic. The most significant limitations include: (1) the high invasiveness of surgical interventions, which increases the potential for morbidity and mortality, (2) the disruption of normal surrounding nerves that innervate visceral organs, which negatively impacts physiological functions (e.g., blood perfusion, muscle contraction and glandular secretion), and (3) the potential activation of compensatory neuroplasticity mechanisms associated with increased neurotransmitter release from residual nerve fibers and the transdifferentiation of cancer cells [72,199,203]. Therefore, it will be necessary to find and design surgical approaches that are significantly less invasive and to find ways to deliver chemical denervating substances directly to the tumor tissue without negative effects on the surrounding normal tissue supplying nerve fibers. Several promising denervation strategies have been indicated as less invasive and more specific, including microsurgical approaches, fiber-specific ablation techniques (e.g., optogenetic and chemogenetic tools), and imaging-based nerve mapping. These techniques allow visualization of nerve distribution within tumors, guiding intervention and selective ablation of tumor-supportive nerves [201,204,205,206]. However, to disrupt complex neuro-tumoral interactions and reduce several adverse side effects associated with denervation procedures, it seems effective to integrate fiber-specific and imaging-guided techniques, as well as combine nerve ablation with therapies targeting neurotrophic factors or axon guidance molecules (Table 3).

### 4.2. Administration of Neurotransmitter Receptor Antagonists

In addition to the surgical or chemical ablation of nerve fibers, several experimental studies demonstrated the importance of administering antagonists of receptors for neurotransmitters released from the axon terminals of nerve fibers innervating tumors to reduce their stimulatory effect on tumor growth and metastasis [1,64,207]. Several studies investigated the effects of β-adrenergic receptor (β-AR) antagonists (e.g., propranolol), muscarinic acetylcholine receptor antagonists (e.g., scopolamine, pirenzepine, darifenacin), and neurokinin-1 receptor (NK-1R) antagonists (e.g., aprepitant) on cancer cell proliferation and tumor progression (Table 3).

#### 4.2.1. Administration of β-Adrenergic Receptor Antagonist Propranolol

The administration of β-adrenergic receptor antagonists, such as propranolol, which limits the influence of the sympathoadrenal system on cancer, has been observed to have two primary effects: inhibition of tumor growth and metastatic spread, as well as enhancement of the efficacy of antitumor therapy (Table 3) [105,208]. The significance of testing the efficacy and use of propranolol in clinical practice is supported by several facts. Firstly, propranolol is an inexpensive, clinically approved β-blocker, which has been used in clinical practice for decades in the treatment of coronary artery disease and hypertension, and during this time, the antitumor effects have been shown. Secondly, the pharmacological properties of propranolol have been described in detail. Finally, the use of propranolol outside of approved indications in cancer patients is approved largely without significant problems [22,64,209]. Several experimental and clinical studies evaluating the effects of propranolol on cancer cell activity, tumor growth, and cancer patient survival show a significant degree of inconsistency. This inconsistency is influenced by factors such as the type and subtype of cancer, the dosage, the method of administration, the individual characteristics of the patient, the duration of administration, and the timing of administration initiation [22,26,210]. The anti-tumor effects of propranolol have been tested in experimental in vitro and in vivo conditions, as well as in clinical studies with cancer patients. At the cellular level, Zhang et al. [211] demonstrated the inhibitory effect of propranolol (in a 100 μM concentration) on the proliferation of PC-2 human pancreatic cancer cells through the induction of apoptosis. Using MIA PaCa-2 and BxPC-3 human pancreatic cancer cell lines, Guo et al. [212] demonstrated the inhibitory effect of propranolol (in a 1 μM concentration) on NE-induced metastasis associated with increased levels of VEGF, MMP-2, and MMP-9. Using human gastric cancer cell lines (SGC-7901 and BGC-823), Liao et al. [213] demonstrated that, depending on the concentration used (50, 100, 200 μM), propranolol inhibited cancer cell proliferation via G0/G1 arrest and G2/M arrest, as well as induced apoptosis. Additionally, treating gastric cancer cells with propranolol enhanced their sensitivity to radiotherapy and led to a more significant decrease in cell viability and clonogenic survivability [214]. Finally, propranolol reduced NF-κB levels in gastric cancer cells, leading to a decrease in the expression of VEGF, COX-2, MMP-2, MMP-9, and EGFR [213,214]. A study conducted by Pasquier et al. [215] showed that treating human breast cancer cells with propranolol (in a concentration of 50–100 μM) inhibits their proliferation and neoangiogenesis. Administering propranolol in combination with chemotherapy enhances its inhibitory effect on cancer cell proliferation and neoangiogenesis [215]. Using the human liver cancer cell lines (HepG2 and HepG2.2.15), Wang et al. [216] demonstrated that propranolol (in a dosage of 40 and 80 μmol/L) inhibits cancer cell proliferation, decreases β_2_-AR expression more than β_1_-AR expression, and induces apoptosis. In addition, propranolol treatment also induced S-phase arrest in both liver cancer cell lines [216]. In breast cancer cell lines, Montoya et al. [64] found that propranolol administration, depending on the concentration used (50, 100, or 200 μM), reduced viability and proliferation. Molecular analysis revealed decreased Ki67 protein expression, reduced phosphorylation of mitogenic signaling regulators (p44/42 MAPK, p38 MAPK, JNK, and CREB), and increased phosphorylation of cell survival/apoptosis regulators (AKT, p53, and GSK3β) following propranolol treatment [64]. Similar anti-tumor results have been obtained as part of in vivo animal experiments. In the context of investigating the effects of propranolol on tumor development and progression, Al-Wadei et al. [217] showed that daily administration of propranolol (in a dosage of 0.3 mg/100 g) for eight months significantly inhibited the development and progression of pancreatic cancer in hamsters with ethanol- and nicotine-derived nitrosamine 4-(methylnitrosamino)-1-(3-pyridyl)-1-butanone (NNK)-induced pancreatitis. This effect was mediated by blockade of cAMP-dependent release of EGF and PKA-dependent release of VEGF, as well as downregulation of the α7nAChR via inhibition of cAMP-mediated subunit assembly [217]. A study by Thaker et al. [105] showed that daily propranolol administration (in a dosage of 2 mg/kg) starting 4 days after tumor cell injection completely inhibited isoproterenol-induced neoangiogenesis and growth of mouse ovarian tumors. In mice with induced melanomas, Kokolus et al. [218] showed that daily administration of propranolol (10 mg/kg) for three weeks alongside immunotherapy enhances its effectiveness and extends survival. In a mouse model of breast cancer, Sloan et al. [45] found that administering propranolol via an intrascapular implanted pellet containing 0.5 mg of propranolol for 21 days led to the complete inhibition of metastasis in stressed animals but did not affect primary tumor growth. Similarly, in a mouse breast cancer model, Campbell et al. [59] demonstrated the inhibitory effect of propranolol on stress-induced bone metastasis when propranolol was administered daily per os via drinking water in a dosage of 0.5 g/L. Additionally, long-term propranolol administration to rats (in a dosage of 20 mg/kg) with breast cancer resulted in reduced NE concentrations in tumors, slowing primary tumor growth and metastasis [15]. The effects of propranolol on cancer progression and survival have also been investigated in cancer patients as part of several clinical studies. In a retrospective, cross-sectional study of breast cancer patients, Montoya et al. [64] found that daily administration of propranolol (in dosages ranging from 80 to 320 mg) over a three-week period reduced tumor proliferation (as measured by Ki67 expression in tumor tissue sections) in the early stages of breast cancer. In a population-based cohort study of patients with malignant melanoma, Lemeshow et al. [209] demonstrated that patients who received propranolol within 90 days prior to cancer diagnosis had longer survival times compared to those who did not receive propranolol. Similarly, in a register-based cohort study of patients with bladder cancer, Udumyan et al. [219] that the use of β-blockers 90 days before cancer diagnosis was associated with lower bladder cancer-specific mortality, particularly among patients with locally advanced or metastatic bladder cancer. From a clinical perspective, the supportive effect of propranolol on the efficacy of standard anticancer therapy is particularly significant. In patients with melanoma who received immunotherapy, Kokolus et al. [218] demonstrated that the concomitant administration of propranolol enhances the antitumor effects of immunotherapy and improves clinical outcomes. Similar results were observed in patients with advanced melanoma who received propranolol alongside chemotherapy, which increased its efficacy [220].

Findings of several studies have demonstrated that the inhibitory effects of propranolol depend on the dosage of propranolol. In a retrospective study conducted by Zheng et al. [221] investigating the effect of antihypertensive treatment on the incidence of breast tumors in hypertensive patients has been demonstrated that the risk of developing breast cancer was lowest in patients treated daily with 25–50 mg propranolol compared to the women treated with higher or lower doses. However, there is a wide spectrum of clinical studies that have not reported the dosage of the administered propranolol [209,222]. Based on these incomplete findings, it may be difficult to determine the exact dosage of propranolol. Therefore, a detailed clinical study testing the optimal dosage of propranolol is very needed.

Propranolol is often administered systemically, either by injection or orally. Another option represents topical propranolol administration, for example, in the treatment of superficial tumors (e.g., melanoma), and this method could potentially reduce the adverse effects accompanying systemic administration and increase its concentration in tumor tissue [22,223]. To improve the efficacy of propranolol, its sustained-release dosage form can be used. For example, Younis et al. described the antiproliferative efficacy of propranolol-loaded trehalose-coated liposomes on human melanoma cells and demonstrated its good cytotoxicity and increasing cancer cell apoptosis. To improve the efficacy of propranolol, its sustained-release dosage form can be used. For example, Younis et al. [224] described the antiproliferative efficacy of propranolol-loaded trehalose-coated liposomes on human melanoma cells and demonstrated its good cytotoxicity and increasing cancer cell apoptosis. A study conducted by Zhou et al. [225] investigated the effectiveness of propranolol liposomes in prostate cancer, pancreatic cancer, and melanoma. They showed that propranolol liposomes had higher cytotoxicity on prostate, pancreatic, and melanoma cancer cells compared to standard propranolol administration and that, in combination with chemotherapy, more effectively restricted the growth of tumors in experimental animals. However, the authors highlighted the fact that combined therapy has been more effective than any single treatment method. Despite limiting several limitations associated with standard propranolol administration, these findings again indicate the significance of propranolol treatment as a supportive, not primary treatment approach.

In the context of the duration of the propranolol administration, results of clinical studies show that long-term use of propranolol is better compared with using it for shorter periods. The relationship between the duration of propranolol administration and the survival of breast cancer patients was monitored in a cohort study conducted by Scott et al. [226], where no effect on patient survival was observed during the first three months of propranolol use, but long-term use of propranolol for more than three years was associated with increased survival in breast cancer patients. Similarly, the results of a population-based cohort study conducted by Chang et al. [227] showed the most substantial protective effect of propranolol against developing head and neck, gastric, colorectal, and prostate cancers in the case of exposure duration exceeding 1000 days.

To investigate the effect of propranolol usage on the prognosis of patients with different molecular subtypes of breast cancer, Løfling et al. [222] conducted a cohort study, the results of which showed an association between propranolol use and increased survival only in patients with triple-negative breast cancer, but not in patients with other breast cancer subtypes. In the context of the type and subtype of cancer, available data suggests that the effectiveness of propranolol is influenced by the tumor localization and implies a dependency on sympathetic nerve fibers in the tumor, which is based on the physiological specializations of the given organ. Therefore, the importance of blocking adrenergic signaling will be more significant in tumors, in which sympathetic nerve fibers play a substantial role (e.g., breast cancer and early stages of prostate cancer), compared to gastrointestinal malignancies, where a dominant role plays cholinergic signaling [1,13]. The biological reason behind the differences in propranolol effectiveness among cancer subtypes remains unclear. However, there has been a suggested importance of immunosuppression in the tumor microenvironment, and that more immunogenic cancer subtypes (e.g., triple-negative breast cancer) may be more sensitive to restoration of anticancer immunity by treatment [222].

Another significant limitation of propranolol use is the need for appropriate timing of administration initiation. In that way, several findings point to the fact that the administration of propranolol appears to be significant in the perioperative period for reducing the negative effects of surgery stress on tumor micrometastasis, neoangiogenesis, and/or stimulation of dormant tumor growth [228,229]. Using a mouse model of ovarian cancer, Lee et al. [228] found that daily administration of propranolol (in a dosage of 2 mg/kg) seven days prior to surgery reduced the stimulatory effect of the stress response activated by surgery on neoangiogenesis and tumor growth. In a randomized controlled trial, Zhou et al. [229] demonstrated that, in women who underwent modified radical mastectomy for treatment of breast cancer, receiving propranolol (20 mg) three times per day from the day of surgery until the third postoperative day suppressed the increase of regulatory T cells and limited the associated inhibition of CD4^+^ T cells in response to increased catecholamine levels due to surgery. A preliminary study of ovarian cancer patients conducted by Jang et al. [230] demonstrated that daily administration of propranolol two days prior to surgery (at a dosage of 40 mg/day) and then through postoperative day seven (at a dosage of 80 mg/day) significantly decreased CA 125 levels, suggesting the potential to decrease perioperative tumor growth. Despite the limitations of the aforementioned studies, such as the small sample size, differences in the timing of propranolol administration, and the short duration of the studies, the effectiveness of perioperative propranolol treatment has been highlighted. However, further studies are needed to determine the optimal time to start propranolol treatment.

Finally, individual characteristics such as health status and personality type may significantly influence the efficacy of propranolol treatment in cancer patients. For instance, elderly patients with multiple comorbidities may experience various side effects from propranolol treatment, which could alter its effect on tumors and reduce patient compliance. Additionally, propranolol is expected to be especially effective in individuals exposed to chronic stress, including those who are newly diagnosed or socially isolated [22,209,231].

Regarding the study of the effectiveness of propranolol administration, the second phase of an interventional clinical trial (NCT04848519) is testing and monitoring the combination of propranolol with immunotherapy in bladder cancer patients. In addition, several other clinical trials testing the effectiveness of propranolol in the perioperative period (NCT06775080, NCT06839144, NCT06145074) and in combination with chemotherapy (NCT05979818, NCT07125391) are in the recruitment phase.

#### 4.2.2. Administration of Cholinergic and Substance P Receptors Antagonists

Unlike the relatively consistent evidence for the stimulatory effects of sympathoadrenal mediators, the effects of acetylcholine on tumor growth depend on tumor type and the expression of cholinergic receptors. Therefore, targeting cholinergic signaling yields conflicting results. For instance, Magnon et al. [1] discovered that the administration of non-selective and selective muscarinic receptor (MR) antagonists (scopolamine and pirenzepine) or the knockout of the M1R gene inhibited metastasis in a mouse model of prostate cancer. Furthermore, the inhibition of gastric cancer cell proliferation was observed following the administration of the M3R antagonist darifenacin in combination with chemotherapy [13]. However, as mentioned above, tumor innervation does not always stimulate tumor growth. In addition, use of cholinergic receptor antagonists is also complicated by the lack of clinical data and the potential for systemic side effects on the heart or cognitive functions. Therefore, it will be necessary to test and assess the effects of acetylcholine receptor antagonists among different cancer types experimentally, and then clinically, to identify and eliminate potential adverse effects (Table 3).

In the context of blocking neurotransmitter signaling, another promising strategy has been suggested by a study conducted by AlAlikhan et al. [207], in which the effects of the antagonist of NK-1 receptors for substance P, aprepitant, on the viability, inflammation, and apoptosis of human ovarian cancer cells were demonstrated. Aprepitant is an FDA-approved medication to prevent chemotherapy-induced nausea and vomiting, as well as postoperative nausea. In the study, treating ovarian cancer cells with aprepitant reduced their viability, downregulated the expression of anti-apoptotic genes, and reduced reactive oxygen species generation [207]. Despite the antitumor effect of aprepitant, further research is recommended to determine its exact effects on various signaling pathways associated with ovarian cancer and to test and evaluate its effects on other cancer types (Table 3).

### 4.3. Administration of Local Anesthetics

Another promising method for reducing the stimulating effect of nerve fibers on tumors is to use local anesthetics, antibodies against neurotrophic factors, or Trk inhibitors. These agents inhibit the transmission of signals, in the form of action potentials, from nerve fibers to tumors, as well as counteracting the neoaxonogenic effects of neurotrophic factors [6,12,26]. However, the effects of local anesthetics on tumor growth are limited by the need for delivery to an area near the tumor or directly to the tumor itself, as well as the short duration of their action. Regarding antibodies against neurotrophic factors or Trk inhibitors, a significant limitation of these approaches is their negative impact on normal neurotrophic factor production and action in the nervous system. Therefore, a targeted application method is needed for both approaches to deliver the agents directly to tumor tissue while preventing them from affecting surrounding tissue structures. An experimental study carried out by Xing et al. [232] showed that incubating the local anesthetic lidocaine (in a concentration of 5 mM) with human liver cancer cells (HepG2 cells) resulted in the suppression of proliferation and the induction of apoptosis of these cancer cells. Similar results have been demonstrated in the study conducted by Chen et al. [108], in which lidocaine was incubated with human melanoma A375 cells, resulting in the inhibition of their proliferation and the arrest of cell-cycle progression in the G1 phase. This was associated with the inhibition of extracellular signal-regulated kinase (ERK) phosphorylation [108]. Incubation of human melanoma cancer cell lines (A375 and Hs294T) with 2% lidocaine and 0.75% ropivacaine resulted in cancer cell viability and an increase in the expression of pro-apoptotic factors (caspase-3 and -8) after 72 h of exposure [233]. Additionally, Castelli et al. [234] demonstrated that incubating the human breast cancer cell line (MDA-MB-231) and the melanoma cell line (A375) with levobupivacaine or ropivacaine resulted in decreased cell viability and increased apoptosis. The effects of local anesthetics on cancer progression have also been tested in experimental animal models. In a mouse model of liver cancer, Xing et al. [232] found that administering lidocaine at a dosage of 30 mg/kg twice per week for 30 days inhibited tumor growth. Furthermore, concurrent administration of lidocaine and cisplatin (3 mg/kg) increased sensitivity to cisplatin [232]. Chen et al. [108] demonstrated in a mouse model of melanoma that three intravenous injections of lidocaine (in a dosage of 100 mg/kg) for one week period reduced tumor growth. In our study, we demonstrated that applying a combination of the local anesthetics 7% lidocaine and 7% tetracaine topically to the tumor surface significantly inhibited the growth of mouse melanomas and enhanced the efficacy of concomitant immunotherapy [16]. However, it remains unclear to what extent the observed effect was due to the influence on nerve signaling or the direct action of the anesthetics on cancer cells.

The use of local anesthetics based on experimental and, to a limited extent, clinical results, shows promising potential for reducing the negative effects of tumor-innervating nerve fibers. Based on current data, the use of local anesthetics in cancer treatment appears to be effective for both acute and chronic use. Acute use of local anesthetics eliminates the effects of surgical stress, which stimulates processes associated with the development of micrometastasis. Chronic use may contribute to reducing tumor growth [16,108]. However, the administration of local anesthetics is accompanied by several issues. First, their effects are limited to the tumor site or its vicinity. This makes them appropriate for the supportive treatment of superficial tumor types (e.g., melanoma), where local anesthetics can be applied repeatedly over a long period of time [22,108]. For other cancer types, it will be necessary to develop targeted application methods directly to the tumor site. Another significant limitation of using local anesthetics in cancer patients is their short-term effects and the risk of potential toxicity or interactions with chemotherapy. Therefore, further research is needed to evaluate the adverse effects associated with the administration of local anesthetics. Currently, phase 3 of the clinical trial (NCT04065009) is ongoing and testing the intraperitoneal administration of local anesthetics in the perioperative period for ovarian cancer patients.

### 4.4. Administration of Antibodies Against Neurotrophic Factors and Tyrosine Kinase Receptor Inhibitors

Among the antibodies against neurotrophic factors, the most important appear to be those directed against nerve growth factor (anti-NGF) and brain-derived neurotrophic factor (anti-BDNF), as these two neurotrophic factors are the primary stimulators of both neoaxonogenesis and cancer cell proliferation in tumors [6,117]. For example, it has been demonstrated that human esophageal cancer cells (KYSE30 and KYSE70) and breast cancer cells (MDA-MB-231) express high levels of NGF and its receptor, TrkA. Co-cultivation of esophageal and breast cancer cells with PC12 cells resulted in neoaxonogenesis, and this activity was inhibited by the administration of 1 μg/mL anti-NGF antibody [6,12,122]. Administering an anti-NGF antibody or the TrkA inhibitor K252a to animals with induced breast tumors every three days for the duration of a 17-day experiment resulted in the inhibition of tumor growth and metastasis [117]. Using a mouse model of gastric and pancreatic cancer, it has been demonstrated that treatment with the Trk inhibitor PLX-7486, which targets NGF-TrkA interactions, inhibits tumorigenesis and prolongs animal survival [34,84]. In human liver cancer specimens, Guo et al. [235] demonstrated by immunohistochemistry high levels of BDNF and TrkB receptors expression, which correlated with more advanced tumor stages. Administering 20 μg/mL of the anti-BDNF antibody or 0.1 μM of the TrkB receptor inhibitor K252a reduced the migration of human cancer cells and induced apoptosis [235]. Using a mouse model of breast cancer, Vanhecke et al. [138] demonstrated that tumor growth was inhibited by stimulating cancer cell apoptosis after administering the anti-BDNF antibody. Furthermore, in an animal model of multiple myeloma, intraperitoneal administration of anti-BDNF antibody inhibited neoangiogenesis and tumor growth, as well as increased animal survival [236]. Finally, a clinical trial conducted by Hong et al. [237] demonstrated that the administration of the Trk inhibitor larotrectinib extended the survival of patients with Trk fusion cancer (Table 3).

Like local anesthetics, experimental and limited clinical results of Trk inhibitors and antibodies against neurotrophic factors show promising potential for reducing the negative effects of intratumoral nerve fibers. The most significant limitation of their use is their negative impact on the production and action of neurotrophic factors, which are normally produced by cells of the central and peripheral nervous systems. Before these substances can be used clinically to limit the neoaxonogenesis in tumors and the negative impact of neurotrophic factors on cancer cell activity, it is important to develop a targeted application method that delivers the substances directly to tumor tissue while preventing them from affecting surrounding normal tissue, as well as disrupting nervous system functions [22,26].

### 4.5. Psychotherapy

Several studies indicate the importance of psychotherapeutic interventions that alleviate symptoms of depression, anxiety, and stress and improve the quality of life of cancer patients, conditioned by the simultaneous inhibitory effect on both main components of the body’s stress response, the sympathoadrenal system and the hypothalamic–pituitary–adrenocortical axis [238]. Two independent studies suggest that psychotherapy improves the quality of life for advanced-stage cancer patients and prolongs the survival for early-stage patients who are older and lack family support (Table 3) [231,239]. From a clinical perspective, psychotherapy is beneficial for cancer patients, but it is very time-consuming, costly, and its effectiveness can vary significantly depending on the type and stage of cancer, the age of the patients, as well as their personality type [231,238]. Nowadays, there are several ongoing clinical trials monitoring psychotherapy effects in cancer patients (NCT06674499, NCT06674499, NCT05385965), as well as several trials in the recruiting phase (NCT04537936, NCT04452825, NCT06200155, NCT07236021).

### 4.6. Heart Rate Variability Biofeedback

Another indicated way to reduce the negative effects of stress response activation on the body is to stimulate the activity of the parasympathetic branch of the autonomic nervous system using the heart rate variability biofeedback (HRV-B) method. This method involves learning to consciously regulate physiological functions, such as heart rate and breathing rate, to achieve a rhythmic diaphragmatic breathing pattern that increases heart rate variability [240,241]. During HRV-B, a sensor is attached to the body surface (e.g., the earlobe) to record physiological functions and is connected to a display device (e.g., smartphone, computer) that provides real-time information about the value of the monitored biological variable, enabling training in its conscious modulation [241].

Heart rate variability (HRV) reflects the dynamic interactions between the neural (sympathetic, parasympathetic, and intracardial nervous system) and humoral influences that regulate cardiac function [242,243]. Clinically, HRV serves as an indicator of the autonomic nervous system’s regulatory flexibility in modulating cardiac activity and thus reflects the level of stress, the presence and prognosis of various diseases (e.g., cardiovascular, inflammatory, neurological, autoimmune, and cancer), treatment effectiveness, the degree of physical stress, and the body’s recovery rate [22,243,244]. Studies evaluating the significance of HRV in cancer patients have shown a strong association between HRV parameters and several prognostic factors, including tumor stage, prostate-specific antigen levels, serum cytokine concentrations, and overall survival (Table 3) [245,246]. Multiple studies involving patients with various oncological diagnoses have demonstrated an association between low HRV and cancer progression or reduced survival. Conversely, high HRV has been associated with a better prognosis for cancer patients [247,248,249]. The scoping review by Spada et al. [241] analyzes evidence on HRV-B effects in thyroid, lung, brain, colon, and hematologic cancers, as well as in survivors or terminal cancer patients. The results of the analyzed studies generally suggest positive effects of HRV-B, including increased HRV values, improved sleep quality, reduced fatigue, stress, and depression, and alleviated pain in patients compared with controls [241]. Although the heterogeneity of reported studies makes it difficult to generalize the effectiveness of HRV-B. In addition, a longitudinal randomized controlled trial conducted by Dolgilevica et al. [250] with primary breast cancer survivors demonstrated that four weeks of remote, smartphone-based HRV-B significantly increased low-frequency HRV and improved sleep quality, cognitive function, fatigue, stress-related symptoms, and anxiety. These data indicate that HRV-B is a feasible intervention for cancer patients and cancer survivors to address diverse chronic symptoms associated with cancer diagnosis, treatment, and post-treatment life. In the context of HRV-B, two clinical studies are currently recruiting participants: one with bladder cancer patients (NCT06269536) and another with colorectal and breast cancer patients (NCT06281145).

**Table 3:** An overview of experimental and clinical approaches aimed at reducing the influence of nerve fibers on cancer.

Intervention Type	Type of Cancer	Mechanism of Action	Experimental and Clinical Results	Studies
**Surgical Denervation**	Prostate Breast Gastric	Surgical cut of the nerves that innervate the tissue of an organ with malignant changes	↓ tumor development and growth in mice ↓ tumor incidence in patients	[1,13,61,198]
**Chemical Denervation**	Fibrosarcoma Colorectal Prostate	Destruction of sympathetic nerve endings induced by the application of 6-OHDA	↓ tumor development and metastasis in mice	[1,4,199]
	Prostate Gastric Fibrosarcoma Liver	Inhibition of acetylcholine release from synaptic vesicles of axonal endings at neuromuscular junctions induced by the application of botulinum toxin	↓ tumor development and growth in mice ↑ effectiveness of radiotherapy and chemotherapy in mice ↑ cancer cell apoptosis in patient tissue samples	[13,198,200]
**β-adrenergic Receptor Antagonist** **(Propranolol)**	Breast Melanoma Ovarian Pancreatic Gastric Bladder	Binding to β_1_- and β_2_-AR and blocking the effects of epinephrine and NE on the processes triggered by adrenergic signaling	↓ neoangiogenesis, tumor growth, and metastasis in mice ↓ human cancer cell proliferation and metastasis ↑ patient survival and potentiation of concurrently administered chemotherapy or immunotherapy	[45,64,105,211,212,213,215,218,219,228]
**Muscarinic Receptor** **Antagonists**	Prostate Gastric Colorectal	Blocking the effects of acetylcholine on tissue cells through binding to muscarinic receptors (for example non-selective antagonist scopolamine, the M1R antagonist pirenzepine, and the M3R antagonist darifenacin)	↓ cancer cell proliferation, tumor growth and metastasis in mice	[1,4]
**Local Anesthetics** **(Lidocaine, Tetracaine,** **Levobupivacaine and Ropivacaine)**	Melanoma Liver Breast	Reversible ↓ of axonal terminal excitation and nerve transmission through the binding and inactivation of voltage-gated Na^+^ channels in the axonal membrane, which induces the ↓ of action potential propagation	↓ cancer cell proliferation and ↑ apoptosis in human ↑ cancer cell apoptosis, ↓ cancer cell proliferation and tumor growth in mice ↓ tumor growth and potentiation of immunotherapy in mice	[16,108,232,234]
**Antibodies Against** **Neurotrophic Factors** **(Anti-NGF, Anti-BDNF)**	Breast Esophageal Liver Multiple myeloma	Antibodies bind to released neurotrophic factors, preventing their association with Trk and p75^NTR^ receptors.	↓ cancer neoaxonogenesis in human ↓ neoangiogenesis, tumor growth and metastasis in mice ↑ cancer cell apoptosis in mice	[12,117,138,235,236]
**NK-1R** **Receptor Antagonist (Aprepitant)**	Breast Ovarian	Blocking the effects of SP on tissue cells through binding to NK-1R	↓ cancer cell viability, ↓ levels of ROS and ↑ cancer cell apoptosis in human	[207,251]
**Trk Inhibitors** **(K252a, PLX-7486, GZD2202, Larotrectinib)**	Gastric Pancreatic Neuroblastoma Head and neck Thyroid Breast	Blocking the effects of neurotrophic factors on tissue cells through binding to Trk receptors	↓ neoaxonogenesis, cancer cells proliferation, cancer development, progression and metastasis in mice ↑ patient survival time	[34,84,237,252]
**Psychotherapy**	All cancer types	Relieve symptoms of depression, anxiety, and stress by inhibiting both the SAM and HPA systems	↑ quality of life and survival time in patients	[231,238]
**HRV Biofeedback**	Prostate Lung Pancreatic Breast Liver	Training-induced conscious stimulation of PNS activity causes the body to calm down, which is associated with increased heart rate variability	Association of ↓ HRV with cancer stage, ↑ levels of PSA and cytokines, and ↓ patient survival	[241,245,246]

↑—stimulatory effects; ↓—inhibitory effects. Abbreviations: 6-OHDA—6-hydroxydopamine; β-AR—β-adrenergic receptors; HPA—hypothalamic-pituitary-adrenocortical system; HRV—heart rate variability; MR—muscarinic acetylcholine receptors; Na^+^ channels—voltage-gated sodium channels; NE—norepinephrine; NK-1R—neurokinin receptor 1; p75^NTR—^common receptor for neurotrophic factors; PNS—parasympathetic nervous system; ROS—reactive oxygen species; SAM—sympathoadrenal system; SP—substance P; Trk—tropomyosin receptor kinase.

## 5. Future Directions of the Research

Research on the role of autonomic and sensory nerve fibers in cancer development and progression contributes significantly to a better understanding of cancer biology. Over the past two centuries, neuro-cancer interactions have been described in detail, and based on this information, a wide range of new therapeutic approaches that could be effective in potentiating standard anticancer therapy have been indicated. However, prior to their use in clinical practice, several issues and unanswered questions must be addressed. For example, although the tumor-promoting and tumor-inhibiting effects of parasympathetic and sensory nerve fibers have been demonstrated, it is still unclear which factors determine whether innervation stimulates or inhibits cancer progression. Regarding parasympathetic nerve fibers, which densely innervate the gastrointestinal tract, it is assumed that their effects on tumors reflect their functional specialization. For this reason, their influence appears to be most significant in gastric and colorectal cancers. Additionally, emerging evidence suggests that parasympathetic nerve fibers may play an important role in tumor progression, particularly through cholinergic anti-inflammatory pathways. Except for the tumor type, localization, and the physiological specialization of nerve fibers, other important intratumoral nerve fiber-determining factors have been suggested, including the type of receptors expressed by cells in the tumor microenvironment and the degree of immunosuppression in tumors. However, to clarify which factors influence the effects of parasympathetic and sensory nerve fibers on tumor progression, longitudinal and tumor-type-specific studies will be required, as well as tools that can manipulate one neural subtype at a time. In that way, spatial omics technology provides a new perspective for analyzing neuro-tumor interactions. For example, spatial transcriptomic counting the number of gene transcripts at distinct spatial locations in a tissue allows for the precise localization of the exact distribution of nerve fibers in the tumors. In combination with single-cell nuclear RNA sequencing, analyzing the transcriptional diversity of many cells in the tumor microenvironment could help us better understand and identify other nerve–tumor mechanisms activated during tumor development and growth.

Another problem is the ambiguity of the results of clinical studies focused on evaluating the antitumor effects of propranolol. Therefore, future clinical trials and studies focusing on specific cancer types, patient populations, and treatment regimens are needed to better define propranolol’s role as an adjunctive therapy. Another problematic approach is the use of methods that limit the effects of intratumoral nerve signaling, such as denervation, local anesthetics, antibodies against neurotrophic factors, and Trk inhibitors. To implement these approaches, it will be necessary to develop a methodology that enables targeted delivery to tumor tissue and influences only the activity of nerve fibers that innervate the tumor microenvironment, while preserving those that innervate normal tissue.

The most significant challenge in studying nerve–tumor interactions is the absence of experimental models that completely replicate human tumor-nerve-immune circuitry. Standard cell culture systems or co-culture models using neurons lack physiological components of the tumor microenvironment and therefore cannot mimic systemic influences (e.g., vascularization, immune components, innervation, absence of brain inputs or endocrine signals). Although in vivo animal models have demonstrated the significance of innervation, these models use young animals without comorbidities or chronic stress, which potentially fails to capture the neuroimmune complexity observed in patients. Furthermore, differences between species (e.g., animal vs. human cells) and the short timescale of animal experiments may prevent the capture of chronic neural remodeling in human cancers.

For future clinical applications assessing the presence and density of nerve fibers in tumors and evaluating the importance of approaches aimed at reducing the negative effects of intratumoral nerve fibers on tumor microenvironment cells, it will be necessary to develop a patient-derived organoid tumor model. This model will allow us to study the significance of different types of innervations and justify the use of nerve-fiber targeted approaches for a given patient. The generation of patient-derived organoid models could be part of personalized anticancer therapy. As part of this therapy, a fresh tumor sample would be collected from the patient to detect the presence and density of different types of innervations in tumors. Based on these results, it will be possible to assess which innervation type plays a prominent role and which could be targeted. The next step is to test and consider the possibility of using supportive therapy and predict its efficacy. Testing results will help identify and evaluate which patients are most likely to benefit from nerve-targeted supportive cancer therapy.

## 6. Conclusions

Accumulating data indicates that the nervous system influences cancer growth and metastasis by releasing neurotransmitters from sympathetic, parasympathetic, and sensory nerve fibers. Tumors promote this process by producing neurotrophic factors, axonal guidance molecules, and exosomes that stimulate neoaxonogenesis. High nerve fiber density is generally considered an unfavorable prognostic indicator, particularly with regard to sympathetic nerve fibers. However, the influence of parasympathetic and sensory fibers is unclear and may differ among tumor types. In some cases, a high density of these fibers is associated with better clinical outcomes. An understanding of the complex interactions between nerve fibers and tumors is essential for introducing new therapeutic approaches and their incorporation into standard therapy, such as administering local anesthetics, propranolol, antibodies against neurotrophic factors, psychotherapy, or heart rate variability HRV biofeedback. Although propranolol administered in combination with chemotherapy has been associated with inhibiting cancer progression, the optimal timing of its administration and the variability of its effects among patients remain issues. The use of local anesthetics and antibodies against neurotrophic factors is limited due to their difficult application. However, procedures that modify neoaxonogenesis and nerve fiber signaling appear to be a promising new therapeutic approach in oncology.

## Data Availability

No new data were created or analyzed in this study. Data sharing is not applicable to this article.

## Abbreviations

The following abbreviations are used in this manuscript:

6-OHDA	6-hydroxydopamine
AGM	Axonal guidance molecules
Akt (PKB)	Protein kinase B
Alkbh5	RNA demethylase AlkB homolog 5
ANS	Autonomic nervous system
nAChRs	nicotinic acetylcholine receptors
ARG-1	arginase-1
ASCL1	Achaete-scute homolog 1
β-AR	β-adrenergic receptors
CAMK4	Calcium/calmodulin-dependent protein kinase 4
cAMP	Cyclic adenosine monophosphate
CCL	Chemokine ligand
CDK	Cyclin-dependent kinase
CGRP	Calcitonin gene-related peptide
CNS	Central nervous system
COA6	Cytochrome c oxidase assembly factor 6
COX-2	Cyclooxygenase-2
CREB	cAMP-responsive element binding protein
CXCL	C-X-C motif chemokine ligand
DCC	Deleted in colorectal cancer
DCX	Doublecortin
DRG	Dorsal root ganglion
EGF	Epidermal growth factor
EGFR	Epidermal growth factor receptor
EMT	Epithelial-mesenchymal transition
EPH	Ephrin receptor
EPI	Epinephrine
ERK 1/2	Extracellular signal-regulated kinase 1/2
FAK	Focal adhesion kinase
GABA	γ-aminobutyric acid
G-CSF	Granulocyte colony-stimulating factor
GDNF	Glial cell line-derived neurotrophic factor
GFRα1	Glial cell line-derived neurotrophic factor family receptor α1
GPI	Glycosylphosphatidylinositol
GSK-3	Glycogen synthase kinase-3
GTPase	Guanosine triphosphatase
HDAC2	Histone deacetylase 2
HIF-1α	Hypoxia-inducible factor-1α
HO-1	Heme-oxygenase-1
HPA	Hypothalamic–pituitary–adrenocortical axis
HPV	Human papillomavirus
HRV	Heart rate variability
HRV-B	Heart rate variability biofeedback
IFC	Immunofluorescence
IFN	Interferon
IHC	Immunohistochemistry
IL	Interleukin
JAK2	Janus kinase 2
KLF4	Krüppel-like factor 4
LIF	Leukemia inhibitory factor
LKB1	Liver kinase B1
m^6^A	N^6^-methyladenosine modifications
MAPK	Mitogen-activated protein kinase
M-CSF	Macrophage colony-stimulating factor
MDSCs	Myeloid-derived suppressor cells
MIC-1	Macrophage inhibitory cytokine-1
miRs	Micro ribonucleic acid
MMP	Matrix metalloproteinase
MNNG	N-methyl-N′-nitro-N-nitrosoguanidine
MR	Muscarinic acetylcholine receptor
MUC-4	Mucin-4
Na^+^	Voltage-gated sodium channels
NE	Norepinephrine
NEO	Neogenin
NEPC	Neuroendocrine prostate cancer
NF-κB	Nuclear factor-κB
NGF	Nerve growth factor
NGL	Netrin-G ligand
NK cells	Natural killer cells
NK-1R	Neurokinin-1 receptor
NMDAR2D	N-methyl-D-aspartate glutamate receptor subunit 2D
NPCs	Neural progenitor cells
NPY	Neuropeptide Y
NPYR	Neuropeptide Y receptor
NRPs	Neuropilins
NT3	Neurotrophin-3
NT4/5	Neurotrophin-4/5
NTNs	Netrins
p75^NTR^	Common receptor for neurotrophic factors
PanIN	Pancreatic intraepithelial neoplasia
PAR1	Protease-activated receptor 1
PC12	Pheochromocytoma cell line
PDX	Patient-derived xenografts
PGE2	Prostaglandin E2
PGP9.5	Protein gene product 9.5
PI3K	Phosphatidylinositol 3-kinase
PJA2	Praja ring finger ubiquitin ligase 2
PKA	Protein kinase A
PLXN	Plexins
PNI	Perineural invasion
PNS	Peripheral nervous system
POU5F1	POU class 5 homeobox 1
proNGF	Nerve growth factor precursor
PTEN	Phosphatase and tensin homolog
RANKL	Receptor activator for nuclear factor κB ligand
RAMP	Receptor activity-modifying protein
RET	Rearranged during transfection receptor tyrosine kinase
Robo	Roundabout receptors
ROS	Reactive oxygen species
SAM	Sympathoadrenal system
SEMA	Semaphorins
siRNAs	Small interfering RNAs
Smad3	Mothers against decapentaplegic homolog 3
SP	Substance P
ssRNA	Single-stranded ribonucleic acid
STAT3	Signal transducer and activator of transcription 3
TACR1	Tachykinin receptor 1
TGF-β	Transforming growth factor-β
TH	Tyrosine hydroxylase
TLR7	Toll-like receptor 7
Trk	Tropomyosin receptor kinase
TSP-1	Thrombospondin 1
UNC5	Uncoordinated 5 homologs
USP33	Ubiquitin-specific protease 33
VAChT	Vesicular acetylcholine transporter
VEGF	Vascular endothelial growth factor
